# Local and regional food production diversity are positively associated with household dietary diversity in rural Africa

**DOI:** 10.1038/s43016-024-01096-6

**Published:** 2025-01-02

**Authors:** Thanh-Tung Nguyen, Matin Qaim

**Affiliations:** 1 Center for Development Research (ZEF), University of Bonn, Bonn, Germany; 2 Institute for Food and Resource Economics (ILR), University of Bonn, Bonn, Germany

**Keywords:** Agriculture, Nutrition

## Abstract

Undernutrition and low dietary quality remain widespread issues in Africa. As most rural households in the region are involved in farming, the diversification of own farm production could improve their access to nutritious foods. Here we use representative panel data from six African countries to estimate this effect across different scales. We show that farm production diversity is positively associated with household dietary diversity—yet the average magnitude of the association is small, depends on the specific measure of production diversity and increases with distance from urban centres. In all countries, markets and market access are more important for dietary diversity than own production. Because village-, town- and district-level production diversity are often positively associated with dietary diversity, higher diversity on each individual farm may not be required. The appropriate spatial scale should be considered when designing diversification strategies.

## Abstract (web summary)

Evidence on the effect of own farm diversification on access to nutritious foods and dietary diversity is mixed, with most studies using cross-sectional data from specific settings. An analysis of panel data from six African countries helps map and estimate this association across different scales.

## Main

More than two billion people worldwide suffer from undernutrition and micronutrient malnutrition, largely caused by inadequate access to diverse foods and healthy diets^1–6^. Higher levels of dietary diversity are positively associated with nutrient adequacy, physical and cognitive development, and overall health^7–12^. Households in rural Africa are particularly affected by poor diets and nutritional deficiencies^1,6,13,14^. Many of these rural households depend on farming for their livelihoods. Increasing the diversity of own farm production may help to improve access to nutritious foods and dietary diversity, but the evidence is mixed^15,16^.

The question regarding to what extent farm production diversity (FPD) influences the dietary diversity and nutrition of households and individuals has been analysed extensively in recent years, with data from many countries in Africa, Asia and Latin America^15–26^. The results suggest positive associations in many but not all situations. Furthermore, the magnitudes of the associations tend to be small on average, meaning that farm diversification strategies might have limited potential to improve household diets and nutrition at scale^16^. More knowledge is required regarding the conditions needed for farm diversification to be effective and what other strategies may be helpful.

One drawback of the existing work on links between production diversity and dietary diversity is that most studies analyse the situation in one country or setting, so drawing broader international conclusions is difficult. A second drawback is that most studies use cross-section observational data, meaning that unobserved heterogeneity may bias the results. A third drawback is that almost all available studies analyse food production diversity only at the level of individual farms, whereas production diversity at the village level or at higher spatial scales may also influence household dietary diversity. If local markets exist and farms differ in their production patterns, not every single farm would need to be very diverse for households to have access to diverse nutritious foods. There is only one study that analyses production diversity at a higher spatial scale, namely, village-level production diversity and its association with child diets and nutrition^27^. This study uses data from various countries in Africa and evaluates village-level production diversity with remotely sensed crop vegetation data^27^. While this is an interesting approach, the production of fruits, vegetables and animal-sourced foods—all potentially important elements of diversified diets—is not captured in the remotely sensed crop vegetation data.

Here we address these limitations by using representative panel data with over 89,000 household observations from six African countries—Ethiopia, Malawi, Niger, Nigeria, Tanzania and Uganda. This large and representative sample enhances the external validity of the findings and also allows us to make interesting comparisons. The data stem from the World Bank’s Living Standards Measurement Study—Integrated Surveys on Agriculture (LSMS-ISA). They were collected between 2008 and 2022 and include details of agricultural production, food consumption and other relevant socio-economic characteristics. The LSMS-ISA data do not include individual-level dietary data, meaning that our evaluation of dietary diversity remains at the household level. Production diversity is measured in terms of the number of different crop and livestock species as well as the number of food groups produced. We use panel data regression models to estimate how FPD is associated with household dietary diversity, controlling for confounding factors and time-invariant unobserved heterogeneity (Methods). We also examine the role of markets, asset ownership and food production diversity at higher spatial scales for household dietary diversity.

## Results

Table 1 presents descriptive statistics of household dietary diversity and FPD in the six African countries. The household dietary diversity score (HDDS) counts the number of different food groups consumed by each household over a period of 7 days. A higher HDDS indicates higher dietary diversity and dietary quality. We see some differences between the countries. The highest mean HDDS is observed in Malawi and the lowest, in Ethiopia.

**Table 1:** Household dietary diversity and FPD

	All countries	Ethiopia	Malawi	Niger	Nigeria	Tanzania	Uganda
**Household dietary diversity (all households)**							
HDDS	5.66 (1.75)	4.42 (1.56)	6.24 (1.75)	5.70 (1.67)	5.98 (1.73)	5.91 (1.63)	5.67 (1.67)
HDDS from own production	1.67 (1.62)	1.39 (1.32)	1.70 (1.58)	0.84 (1.06)	1.23 (1.26)	1.69 (1.72)	2.48 (1.79)
HDDS from market purchases	4.50 (2.23)	3.08 (1.97)	5.00 (2.31)	5.27 (1.92)	5.24 (2.00)	4.62 (2.25)	3.98 (2.11)
HDDS from other sources	0.64 (1.02)	0.27 (0.64)	1.22 (1.25)	0.49 (0.91)	0.55 (1.01)	0.65 (0.97)	0.68 (1.02)
Food from own production (percentage of value)	33.14 (33.52)	42.84 (39.47)	31.11 (31.39)	19.48 (27.34)	23.91 (28.44)	31.72 (34.48)	42.58 (32.12)
Food from market purchases (percentage of value)	59.13 (34.64)	50.02 (39.56)	55.89 (33.56)	75.22 (29.72)	69.79 (29.97)	59.78 (35.89)	50.25 (32.11)
Food from other sources (percentage of value)	7.73 (16.85)	7.14 (19.59)	13.00 (20.42)	5.30 (14.37)	6.30 (14.77)	8.49 (16.94)	7.17 (15.49)
Observations	89,742	13,511	9,163	7,046	18,592	21,117	20,313
**FPD (households with own farm production)**							
FPD (species)	5.55 (3.33)	7.38 (4.23)	4.04 (2.33)	4.45 (2.37)	4.33 (2.27)	5.85 (3.81)	6.12 (2.78)
Livestock production diversity (food groups)	3.29 (1.51)	3.84 (1.81)	3.08 (1.42)	2.48 (1.09)	2.59 (1.12)	3.55 (1.64)	3.62 (1.28)
Crop production diversity (food groups)	1.07 (0.88)	1.64 (0.96)	0.76 (0.77)	0.98 (0.64)	0.74 (0.60)	1.18 (1.00)	1.05 (0.81)
FPD (food groups)	2.22 (1.14)	2.20 (1.41)	2.32 (1.09)	1.50 (0.99)	1.85 (0.94)	2.38 (1.21)	2.58 (0.91)
Farms growing non-food cash crops (%)	25.63 (43.66)	47.33 (49.93)	18.59 (38.90)	1.05 (10.21)	6.46 (24.58)	30.19 (45.91)	34.28 (47.47)
Observations	67,221	9,981	7,359	5,312	12,882	14,921	16,766

Mean values are shown with standard deviations in parentheses.

Across the six countries, farms produce around 5.5 different crop and livestock species on average, which is quite high given the small farm sizes. Over 75% of the farms in the sample have a landholding of less than 2 ha. Strikingly, the average number of species produced is highest in Ethiopia and lowest in Malawi—the exact opposite of what we found for household dietary diversity (Table 1). This is an indication that FPD may not be the most important driver of household dietary diversity.

Table 1 also shows that the number of food groups produced by each farm is lower than the number of food groups consumed by the farm households. Households consume significantly more food groups from market purchases than from own production, in spite of the fact that we exclude foods with low nutrient density—such as sugar, oils and fats, condiments and beverages, which are mainly purchased—from the food group calculations. Also, in terms of overall food consumption, market purchases often matter more than own production. Across the six countries, households obtain 33% of the food values consumed from subsistence production.

### Role of FPD in dietary diversity

**Fig. 1:**
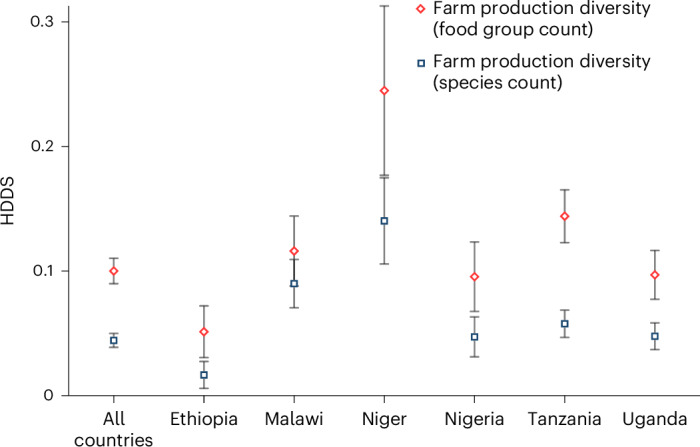
Associations between FPD and household dietary diversity. Coefficient estimates from correlated random-effect models are shown with error bars representing 95% confidence intervals. The models with data from all countries include 89,742 observations. Full model results and control variables included are shown in Supplementary Tables 1 and 2.

We now estimate the associations between FPD and the HDDS with regression models, controlling for possible confounding factors (Methods). We find positive and statistically significant estimates in all cases, but the coefficient sizes are relatively small on average (Fig. 1). When FPD is measured in terms of the number of species produced and data from all six countries are included, the mean coefficient is 0.044 implying that households would have to produce over 20 additional species on their farms to increase the HDDS by one food group. The coefficient sizes are larger when FPD is measured in terms of the number of food groups produced. Here the mean coefficient estimate of 0.10 across the six countries implies that households would have to produce 10 additional food groups to increase the HDDS by one unit. However, the coefficient sizes vary by country. The largest coefficient is observed in Niger and the smallest, in Ethiopia. In the following analysis, we measure FPD only in terms of the number of food groups produced.

### Other factors influencing dietary diversity

**Fig. 2:**
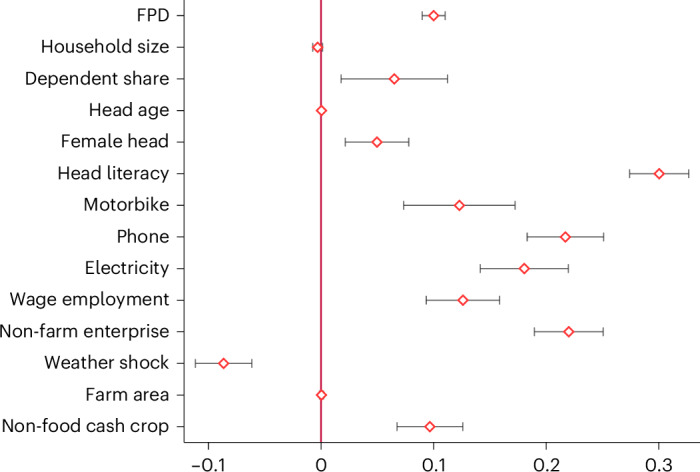
Associations between socio-economic variables and household dietary diversity. The dependent variable is household dietary diversity measured in terms of the HDDS. Coefficient estimates from correlated random-effect models are shown with error bars representing 95% confidence intervals (89,742 observations). Full model results and control variables are shown in Supplementary Table 2. FPD measured in terms of the number of food groups produced.

FPD is not the only factor influencing household dietary diversity. The results in Fig. 2 show that several other socio-economic characteristics matter in the context of rural Africa (a breakdown by country is shown in Supplementary Table 2). Weather shocks—defined as the occurrence of a drought, flood, hurricane or related extreme event over the last 12 months—are negatively associated with the HDDS. Several other socio-economic characteristics are positively associated with the HDDS. The production of non-food cash crops—such as cotton, coffee, tea or tobacco—on own farms seems to contribute to higher household dietary diversity through positive cash income effects. Similarly, off-farm wage employment and self-employment in own non-farm enterprises are positively associated with the HDDS. Education of the household head, measured in terms of literacy, also has a large positive estimation coefficient. These findings are in line with earlier studies on links between socio-economic factors, food security and dietary quality^28–36^.

Asset ownership is positively associated with dietary diversity. Assets such as mobile phones, motorbikes and electricity are commonly used proxies of wealth in the context of rural Africa. In addition, these assets are directly linked to food market access as well as food preparation and storage, so positive associations with dietary diversity are plausible. Finally, female-headed households have higher HDDSs than male-headed households (Fig. 2). Female-headed households are often worse off in terms of wealth and access to land and other productive resources^37^, which might lead to lower welfare and dietary quality. However, in our models, we control for such other factors, which probably explains why we see this positive association here.

### FPD and the role of markets

**Fig. 3:**
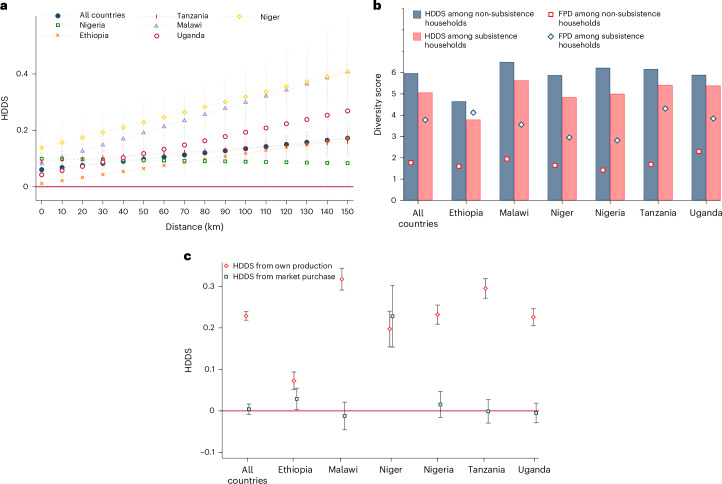
Associations between FPD, markets and household diets. **a**, Associations between FPD and household dietary diversity by distance to the nearest urban centre. Marginal effects from correlated random-effect models are shown with error bars representing 95% confidence intervals. The model with data from all countries includes 61,916 observations. Full model results and control variables included are shown in Supplementary Table 3. **b,** FPD and household dietary diversity in subsistence and non-subsistence households. Subsistence households are defined as those that obtain more than 50% of their food consumption value from own production. The sample with data from all countries includes 78,003 observations. **c**, Associations between FPD and household dietary diversity obtained from own production and market purchases. Coefficient estimates from correlated random-effect models are shown with error bars representing the 95% confidence intervals. The models with data from all countries include 82,550 observations. Full model results and control variables included are shown in Supplementary Tables 4 and 5. FPD was measured in terms of the number of food groups produced.

As seen in Fig. 1, the size of the association between FPD and the HDDS varies between countries. However, the association also varies within countries, depending on infrastructure, market access and possibly other spatial conditions. Distance to urban centres—a proxy for the costs of accessing markets in rural Africa^38^—has a negative association with the HDDS in all six study countries (Supplementary Table 3). In the pooled sample, the average household is located around 31 km away from the next urban centre. In addition, as shown in Fig. 3a, with increasing distance to urban centres, the size of the association between FPD and the HDDS rises. In other words, in remote locations with poor market access, FPD plays a more important role for household diets than in better-connected locations. This is expected given that households in remote areas are often more subsistence oriented.

Figure 3b shows that FPD in subsistence households (those with food consumption shares from own production above 50%) is significantly higher than in non-subsistence households. However, at the same time, the HDDS in subsistence households is significantly lower than in non-subsistence households. This comparison suggests that increasing FPD cannot fully compensate for the negative effect of lower market access on household dietary diversity.

Figure 3c shows that there is also a direct link between FPD and subsistence orientation. FPD seems to encourage stronger subsistence orientation, as the association with HDDS from own production is larger than the association with the HDDS from market purchases. In fact, the association between FPD and the HDDS from market purchases is zero or near zero in all countries, except for Niger. The larger coefficient estimate in Niger suggests that farm diversification there is driven not only by subsistence demand but also by market incentives.

### Local and regional food production diversity

**Fig. 4:**
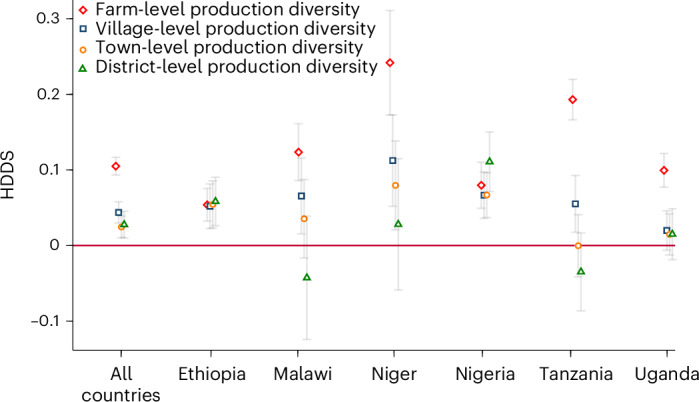
Associations between production diversity at different spatial scales and household dietary diversity. Production diversity is measured in terms of the number of food groups produced at each spatial scale. Coefficient estimates from correlated random-effect models are shown with error bars representing 95% confidence intervals. The models with data from all countries include 65,579 observations. Full model results and control variables included are shown in Supplementary Tables 6–9.

So far, we have looked at the associations between own FPD and the HDDS. Now we want to understand whether local and regional food production diversity—beyond own farms—may also influence household diets and nutrition. Figure 4 summarizes results from various regressions, comparing the coefficient estimates for farm-level production diversity with those for food production diversity at higher spatial scales, namely, village level, town level and district level (Methods). In the models with data from all countries combined, all local and regional metrics of food production diversity have positive and significant associations with HDDS. Also, in the individual country models, most of the local and regional production diversity metrics have positive associations with household dietary diversity. This is plausible because more diverse agricultural production at the local and regional levels also means higher levels of food diversity available in local and regional markets. Above, we have shown that market purchases are more important for the HDDS than own household production.

Comparing production diversity at different spatial scales, we have found that farm-level production diversity has the largest positive associations with the HDDS in most cases, followed by village level and then town and district levels. This is as expected. Strikingly, however, in Ethiopia and Nigeria, the coefficient sizes of district-level production diversity are at par with those of farm-level production diversity or even larger, underlining the crucial role of local and regional markets.

## Discussion

Our study adds to the existing literature on the links between FPD and household diets and nutrition^15–25^ in that we analyse associations with representative panel data from six countries in Africa, namely, Ethiopia, Malawi, Niger, Nigeria, Tanzania and Uganda. We also examine more specifically than earlier work on what factors the size of the associations depend and what roles market access and food production diversity at higher spatial scales may play. Our insights contribute to a better understanding of the complex relationships and can help design strategies for improving rural diets and other important sustainability outcomes. Food system diversification is generally considered a useful approach to enhance sustainability and resilience, but how exactly diversification should look like in specific contexts remains an open question^39,40^.

Our results suggest that FPD is positively associated with HDDS. The size of the association depends on how exactly FPD is measured: it is larger when FPD is measured in terms of the number of food groups produced than when it is measured in terms of the number of crop and livestock species produced. This is plausible, as producing and consuming additional species do not necessarily add to the HDDS if the species belong to the same food group (for example, maize, millet, teff, wheat and sorghum all belonging to the food group ‘cereals’).

However, regardless of how FPD is measured, its mean association with HDDS is relatively small. This is consistent with earlier research^16^. Farms would have to produce many more species and food groups to have a sizeable influence on dietary diversity through this channel. The potentials for drastic farm diversification are limited because most farms in Africa are small and quite diverse anyway. Our results also suggest that farm diversification is often associated with higher levels of subsistence, meaning that very diverse farms may forego development potentials emerging from more market interactions. Markets are important sources of nutritious foods. On average, rural households in all six African countries obtain most food groups from market purchases and only a small fraction from own production. Also, market-oriented households tend to have higher HDDSs than their more subsistence-oriented counterparts.

In spite of small mean coefficient estimates, we find some interesting differences between countries. In Niger, the association between FPD and the HDDS is larger than in the other five countries. Niger is much drier than the other countries and has lower agricultural production potential^41^. Farm diversity in Niger is very low on average; most of the foods are purchased from the market (Table 1). Many farmers in Niger grow cereals with very low average yields. Keeping cattle and small ruminants in pastoral and semi-pastoral systems is also common. Higher production diversity in this context often means that additional livestock species—such as chicken—are kept. Moreover, a few farmers in Niger with access to irrigation grow vegetables and fruits primarily for market sales. Hence, unlike in other countries, in Niger, higher FPD is not associated with subsistence, but with better access to markets and technologies and higher levels of commercialization. This is supported by our finding that FPD in Niger is strongly associated with the HDDS from market purchases (Fig. 3c), which is not the case in the other five countries. These comparisons underline the fact that the effects of FPD depend on the context and that market-led diversification strategies have stronger positive diet and nutrition effects than subsistence-led diversification strategies^16,23,24^.

We also find differences within countries. FPD has larger positive associations with the HDDS in remote rural areas than in settings that are better connected to markets and urban centres. This means that farm diversification strategies may be more effective in improving diets in remote locations. Interestingly, however, we find that village-, town- and district-level food production diversity have positive associations with the HDDS as well. In some cases, these associations are in a similar magnitude as those between farm-level production diversity and the HDDS. More diverse local and regional production means that more diverse foods are available and accessible through local and regional markets. These results suggest that diversification strategies should focus not only on individual farms but also on local and regional food systems more broadly.

In settings with good infrastructure conditions and spatially integrated markets, the diversity of foods sold in local markets may not depend much on the diversity of foods produced locally because foods from other regions can easily be imported. However, this is not the case in many rural regions of Africa. With poor infrastructure conditions, perishable nutritious foods—such as vegetables, fruits and dairy—are not traded much into remote rural regions, unless they are produced locally. Hence, local production diversity is important but does not necessarily mean that every individual farm needs to be extremely diverse. For individual farms, high levels of production diversity may face land and labour constraints, hampering efficiency and possible gains from specialization. Local and regional food production diversity can also be achieved if individual farmers specialize to some extent, as long as local markets exist and not all farmers specialize on the same types of output. Strengthening local markets and supply chains for perishable foods could also create new market incentives for farm diversification, such that diversification would be driven by market opportunities and not primarily by household subsistence needs.

In conclusion, increasing the production diversity of individual farms may be useful in very specific situations but should not be seen as a general strategy to improve dietary quality in rural Africa. In all six countries, market purchases are more important for dietary diversity than own production. Improving market access and the functioning of local markets for nutritious foods should therefore receive higher policy priority. Local and regional production diversity matter, but these are not the same thing as production diversity on individual farms. The appropriate spatial scale needs to be considered when designing food system diversification strategies. Beyond production diversity and food market functioning, access to productivity-enhancing agricultural technologies, infrastructure and non-farm income sources are other important avenues to improve rural diets in Africa, as our results also show.

We acknowledge that there are a few limitations of the data and the approaches used in this study. First, while we use panel data models that allow us to better control for confounding factors than most previous studies, we cannot fully rule out endogeneity bias, so we interpret our estimates as associations, not as causal effects. Second, the nationally representative LSMS-ISA data provide details on household-level food consumption, not on individual-level food intakes. We rely on HDDSs as simple proxies of dietary quality, not considering food quantities, which are also important for nutritional outcomes. Third, links between agricultural production and household food consumption tend to vary seasonally, which is not properly captured in the data. Hence, follow-up studies will be useful to further validate our findings.

## Methods

### Data

We use data from the LSMS-ISA, focusing on six African countries, namely, Ethiopia, Malawi, Niger, Nigeria, Tanzania and Uganda. The LSMS-ISA surveys are nationally representative and conducted by the country statistical offices with support from the World Bank. In all six countries, a minimum of two survey waves were conducted, using a panel structure. In Ethiopia, data from three waves conducted between 2011 and 2016 are available. In Malawi, four waves (2010–2020) are available; in Niger, two waves (2011–2015); in Nigeria, four waves (2010–2019); in Tanzania, five waves (2008–2022); and in Uganda, seven waves (2009–2020) (Supplementary Table 10). LSMS-ISA surveys were also conducted in Burkina Faso and Mali, but in those two countries, no panel data are available, so we decided to only focus on six countries.

In all countries, the data were collected through face-to-face interviews using structured questionnaires, capturing information on farming and other household economic activities, food and non-food consumption, asset ownership and various other socio-economic factors. Food consumption was captured through 7 day recalls at the household level. In some of the countries, certain data were captured more than once per year. In those cases, we arranged the data in a way that makes them comparable to those of other countries as much as possible. All survey waves from the six countries combined include around 90,000 observations. We exclude observations with missing values or outliers for food consumption and other key variables. Our final sample includes 89,742 observations (Supplementary Table 10).

Not all of the households in the sample are involved in farming. In the main analysis, we include all households, as the decision whether or not to farm is endogenous and may change over time. However, in a robustness check, we also run all regressions only for the subsample of households involved in farming. Furthermore, as sample attrition over the various survey waves occurs, we also run all regressions only with the balanced sample and with the full farmer sample but correcting for possible attrition bias. The results of these robustness checks are very similar to the main results and support the same conclusions (Supplementary Tables 11–18).

### Key variables

#### Dietary diversity

Dietary diversity at the household level is measured in terms of the HDDS, which reflects the economic ability of a household to consume a range of food options and their food security status^42,43^. The HDDS typically includes 12 food groups, namely (1) cereals; (2) legumes, nuts and seeds; (3) vegetables; (4) white roots and tubers; (5) fruits; (6) eggs; (7) milk and related products; (8) fish; (9) meat; (10) spices, condiments and beverages; (11) sugar and sweets; and (12) oils and fats. However, the last three food groups are commonly perceived as less nutritious and, if included, might hinder the capacity of the HDDS to properly reflect dietary quality^21,24,43^. We therefore do not include these three food groups and calculate the HDDS only with the remaining nine groups. We calculate the HDDS based on the 7 day food consumption data collected in the LSMS-ISA surveys.

#### FPD

FPD is measured by two indicators, namely (1) the number of species produced by each farm and (2) the number of food groups produced by each farm. The species count is a common and intuitive way of measuring the diversity of farms^15,16,21^. However, producing a larger number of species does not necessarily increase food group diversity, because many relevant species produced by farms belong to the same food group^44^. Maize, sorghum and millet are all cereals. Tomatoes, cabbages and eggplants are all vegetables, just to name a few examples. Hence, recent studies have measured FPD also in terms of the number of different food groups produced^19,23,24,45^. For the calculation, we use the same nine food groups as listed above for the HDDS. We use FPD measured in terms of food groups produced for most of the analysis, but provide an explicit comparison of both metrics’ associations in Fig. 1.

#### Local and regional food production diversity

Food production diversity at the village, town and district levels is measured by counting the number of different food groups produced by farms sampled in the respective villages, towns and districts. Note that the administrative levels have different names across the six study countries. Villages are sometimes called clusters or parishes; towns are sometimes called wards or sub-counties; districts are sometimes called departments, counties or woredas. We decided to use the same terminology across study countries to facilitate comparisons. For some of the observations, exact village classifications were missing. These observations were left out for the analysis in Fig. 4. Furthermore, villages with only one sampled farm household were omitted for this part of the analysis because, in these cases, farm-level and village-level production diversity would be identical. On average, the number of farm households sampled per village is seven across the six countries. Of course, the number of food groups produced by sampled households is only a lower-bound estimate of the total food production diversity at the village level because non-sampled households may possibly produce additional food groups. This is a drawback of our approach. However, an advantage of our approach is that we also cover species and food groups that are difficult to evaluate with remotely sensed data^27^, including fruits, vegetables and animal-sourced foods.

### Regression models

#### Associations between FPD and the HDDS

We estimate associations between FPD and the HDDS with panel data regression models of the following type:

1 $${\rm{HDDS}}_{it}=\alpha +\beta {\rm{FPD}}_{it}+\delta\;{{\bf{X}}}_{it}+\vartheta {\overline{{\rm{FPD}}}}_{i}++\gamma {\overline{X}}_{i}+\theta {{\bf{C}}}_{i}+\tau {{\bf{T}}}_{t}+{\varepsilon }_{it}$$

where subscript *i* indicates households and subscript *t* indicates survey years. **X** is a vector of control variables including asset ownership (farmland, motorbikes and so on), other household farm and socio-economic characteristics (cash crop production, off-farm employment, access to infrastructure and institutions, and so on), characteristics of the household head (sex, age, education) and weather shocks (see Supplementary Table 19 for a full list and descriptive statistics). **C** and **T** are vectors of country and time fixed effects, the former of which is only included in the pooled models with data from all six countries. *α*, *β*, *δ*, *υ*, *γ*, *θ* and *τ* are parameters to be estimated, and *ε* is a random error term. We are particularly interested in the estimate of the coefficient *β*. A positive and statistically significant *β* would indicate that FPD is positively associated with the HDDS.

One concern in estimating such models with observational data is the possible endogeneity of FPD. FPD is probably influenced by many observed and unobserved factors. While controlling for observed heterogeneity is straightforward, controlling for unobserved heterogeneity is sometimes not. The advantage of panel data over cross-sectional data is that we can control for time-invariant unobserved heterogeneity, either through including household fixed effects (FE) or correlated random effects (CRE)^46^. Both the FE and the CRE estimators do not inherently control for time-varying heterogeneity. The FE estimator fully relies on variation within households over time, so coefficients for explanatory variables with limited variation over time can sometimes not be estimated efficiently and time-invariant variables are dropped. As we are also interested in the coefficients of variables with limited variation over time, we use the CRE estimator for the main analysis and include potentially endogenous variables as time averages, indicated by $\overline{{\rm{FPD}}}$ and $\overline{X}$ in equation (1). However, in robustness checks, we also use the FE estimator. In addition, as the HDDS is a count variable, we conduct additional robustness checks with Poisson specifications. Results from the FE and Poisson CRE specifications are shown in Supplementary Tables 20–27. They are similar to the original estimates and support the same conclusions.

In addition to the standard models in equation (1), we also analyse how FPD is associated with dietary diversity from different sources by disaggregating HDDS into (1) HDDS from market purchases and (2) HDDS from own production, using both as outcome variables in separate regressions. Furthermore, to analyse the role of market access and urban proximity, in some of the regressions, we include distance to the nearest urban centre (defined as a city with a population of at least 20,000) and an interaction term between FPD and this distance variable as additional regressors.

#### Role of local and regional production diversity for the HDDS

We estimate associations between local and regional production diversity and the HDDS with regression models of the following type:

2 $${\rm{HDDS}}_{ivt}=\omega +\pi {\rm{LPD}}_{vt}+\varphi {\bf{X}}_{ivt}+\sigma {\overline{\rm{LPD}}}_{v}+\rho {\overline{X}}_{iv}+\psi {N}_{vt}+\varsigma {\bf{C}}_{i}+\Omega {\bf{T}}_{t}+{\varepsilon }_{ivt}$$

where LPD_*vt*_ stands for local (or regional) production diversity at the spatial scale of interest *v*, which can be the village, the town or the district in which household *i* resides. As the number of farmers sampled at each spatial scale differs, we additionally control for the number of sampled farmers, *N*_*vt*_. *ω*, *π*, *φ*, *σ*, *ρ*, *ψ*, *ς* and *Ω* are parameters to be estimated. All other variables in equation (2) are defined as in equation (1).

As explained above, for this part of the analysis, we had to exclude some of the observations. The total number of observations used for estimating equation (2) is 65,579 in the pooled country model. To compare the estimates for FPD and local and regional production diversity at different spatial scales, we use this same smaller sample for all estimates shown in Fig. 4. The coefficients for FPD with this smaller sample hardly differ from the full-sample estimates in Fig. 1, underlining the overall robustness of our findings.

### Reporting summary

Further information on research design is available in the Nature Portfolio Reporting Summary linked to this article.

## Supplementary information

Supplementary Information Supplementary Tables 1–27.

Reporting Summary

## Data Availability

The data used for this analysis are available at https://www.worldbank.org/en/programs/lsms/initiatives/lsms-ISA.

## Code availability

The code used for this analysis is available at 10.7910/DVN/NYPA0Z.
