# Supplementary material for: Local and regional food production diversity are positively associated with household dietary diversity in rural Africa
Source: Nat Food. 2025 Jan 2;6(2):205–12. doi: 10.1038/s43016-024-01096-6 (PMC11850281; doi:10.1038/s43016-024-01096-6)
Supplement: Supplementary file 1 — Supplementary Tables 1–27. [file 43016_2024_1096_MOESM1_ESM.pdf]

nature food

Supplementary information https://doi.org/10.1038/s43016-024-01096-6

Local and regional food production diversity are positively associated with household dietary diversity in rural Africa

In the format provided by the authors and unedited

Supplementary Information

Local and regional food production diversity are positively associated with household dietary diversity in rural Africa

Tables 1-27

Table 1. Associations between farm-level production diversity (species count) and household dietary diversity

*** p<0.01, **p<0.05, * p<0.1, farm-level production diversity measured in terms of species count. The p-values of coefficients are obtained from two-sided z-tests with standard errors clustered at the household level

	Correlated random effects models						
	All	Ethiopia	Malawi	Niger	Nigeria	Tanzania	Uganda
Production diversity	0.044***	0.017***	0.090***	0.140***	0.047***	0.058***	0.048***
	(0.003)	(0.006)	(0.010)	(0.018)	(0.008)	(0.006)	(0.005)
Household size	-0.002	0.051***	-0.012	0.014**	-0.020***	-0.005	0.015***
	(0.002)	(0.007)	(0.009)	(0.006)	(0.004)	(0.004)	(0.005)
Share of dependent members	0.072***	-0.015	-0.333***	-0.039	0.133***	0.099**	0.162***
	(0.024)	(0.054)	(0.073)	(0.096)	(0.049)	(0.051)	(0.055)
Head age	0.001	-0.003***	-0.003***	0.003**	0.005***	-0.001	-0.001
	(0.000)	(0.001)	(0.001)	(0.001)	(0.001)	(0.001)	(0.001)
Head female	0.049***	-0.015	-0.100**	0.108*	0.209***	0.070**	0.067**
	(0.014)	(0.033)	(0.042)	(0.058)	(0.035)	(0.028)	(0.032)
Head literacy	0.300***	0.406***	0.294***	0.340***	0.193***	0.306***	0.266***
	(0.013)	(0.030)	(0.045)	(0.044)	(0.027)	(0.029)	(0.031)
Motorbike	0.124***	-0.124	0.233*	0.246**	0.034	0.184***	0.227***
	(0.025)	(0.132)	(0.137)	(0.097)	(0.038)	(0.059)	(0.050)
Phone	0.219***	0.196***	0.256***	0.093	0.184***	0.307***	0.257***
	(0.017)	(0.037)	(0.053)	(0.074)	(0.038)	(0.040)	(0.034)
Electricity	0.182***	0.153***	0.448***	0.214**	0.088**	0.081*	0.354***
	(0.020)	(0.045)	(0.086)	(0.107)	(0.045)	(0.043)	(0.034)
Wage employment	0.125***	-0.035	0.182***	0.202**	0.208***	0.134***	0.060**
	(0.017)	(0.054)	(0.056)	(0.085)	(0.048)	(0.030)	(0.028)
Nonfarm business	0.219***	0.208***	0.262***	-0.198***	0.275***	0.260***	0.189***
	(0.016)	(0.047)	(0.041)	(0.065)	(0.039)	(0.032)	(0.028)
Weather shock	-0.081***	-0.167***	-0.192***	-0.133***	0.006	-0.149***	-0.017
	(0.013)	(0.029)	(0.033)	(0.045)	(0.047)	(0.027)	(0.024)
Farm land area	0.001	0.008**	0.014	-0.001	-0.007	0.002	0.001
	(0.001)	(0.003)	(0.026)	(0.002)	(0.006)	(0.002)	(0.001)
Non-food cash crop	0.065***	0.223***	-0.055	0.149	0.331***	-0.063**	-0.052*
	(0.016)	(0.033)	(0.048)	(0.220)	(0.060)	(0.031)	(0.028)
Motorbike (time average)	0.074**	1.150***	0.325	0.107	-0.011	0.486***	0.227***
	(0.034)	(0.215)	(0.212)	(0.119)	(0.053)	(0.076)	(0.080)
Phone (time average)	0.543***	0.523***	0.463***	0.338***	0.802***	0.402***	0.398***
	(0.025)	(0.056)	(0.076)	(0.092)	(0.059)	(0.054)	(0.060)
Electricity (time average)	0.682***	0.625***	0.729***	0.524***	0.794***	0.547***	0.469***
	(0.027)	(0.067)	(0.110)	(0.125)	(0.058)	(0.055)	(0.064)
Wage employment (time average)	0.235***	0.481***	0.382***	0.202*	0.217***	0.119***	0.147***
	(0.025)	(0.081)	(0.079)	(0.104)	(0.064)	(0.042)	(0.053)
Nonfarm business (time average)	0.186***	0.088	0.338***	0.460***	-0.023	0.238***	0.348***
	(0.022)	(0.060)	(0.068)	(0.081)	(0.051)	(0.044)	(0.051)
Production diversity (time average)	-0.021***	0.001	-0.056***	-0.156***	-0.073***	-0.029***	-0.001
	(0.004)	(0.007)	(0.014)	(0.020)	(0.010)	(0.007)	(0.008)
Constant	4.486***	3.226***	5.324***	4.573***	4.644***	4.380***	4.003***
	(0.046)	(0.067)	(0.086)	(0.105)	(0.074)	(0.062)	(0.080)
Year and country dummies	yes	yes	yes	yes	yes	yes	yes
No. of obs.	89742	13511	9163	7046	18592	21117	20313
P-value	0.000	0.000	0.000	0.000	0.000	0.000	0.000

Table 2. Associations between farm-level production diversity (food groups) and household dietary diversity

*** p<0.01, **p<0.05, * p<0.1, farm-level production diversity measured in terms of food groups. The p-values of coefficients are obtained from two-sided z-tests with standard errors clustered at the household level

	Correlated random effects models						
	All	Ethiopia	Malawi	Niger	Nigeria	Tanzania	Uganda
Production diversity	0.100***	0.051***	0.116***	0.245***	0.095***	0.144***	0.097***
	(0.005)	(0.011)	(0.014)	(0.035)	(0.014)	(0.011)	(0.010)
Household size	-0.003	0.048***	-0.012	0.014**	-0.025***	-0.004	0.015***
	(0.002)	(0.007)	(0.009)	(0.006)	(0.004)	(0.004)	(0.005)
Share of dependent members	0.065***	-0.024	-0.338***	-0.034	0.137***	0.089*	0.150***
	(0.024)	(0.054)	(0.073)	(0.096)	(0.049)	(0.051)	(0.055)
Head age	0.000	-0.003***	-0.003**	0.003**	0.005***	-0.001	-0.002
	(0.000)	(0.001)	(0.001)	(0.001)	(0.001)	(0.001)	(0.001)
Head female	0.050***	-0.009	-0.100**	0.112*	0.221***	0.070**	0.067**
	(0.014)	(0.033)	(0.042)	(0.058)	(0.035)	(0.028)	(0.032)
Head literacy	0.300***	0.406***	0.293***	0.347***	0.197***	0.311***	0.266***
	(0.013)	(0.030)	(0.045)	(0.044)	(0.027)	(0.029)	(0.031)
Motorbike	0.123***	-0.138	0.249*	0.242**	0.038	0.168***	0.227***
	(0.025)	(0.132)	(0.137)	(0.096)	(0.038)	(0.059)	(0.050)
Phone	0.217***	0.192***	0.257***	0.098	0.185***	0.301***	0.254***
	(0.017)	(0.037)	(0.053)	(0.075)	(0.038)	(0.040)	(0.034)
Electricity	0.180***	0.147***	0.453***	0.231**	0.088**	0.079*	0.339***
	(0.020)	(0.045)	(0.086)	(0.106)	(0.045)	(0.043)	(0.034)
Wage employment	0.126***	-0.034	0.176***	0.221***	0.212***	0.131***	0.061**
	(0.017)	(0.054)	(0.056)	(0.084)	(0.048)	(0.030)	(0.028)
Nonfarm business	0.220***	0.212***	0.260***	-0.178***	0.278***	0.258***	0.194***
	(0.016)	(0.047)	(0.041)	(0.066)	(0.039)	(0.032)	(0.028)
Weather shock	-0.087***	-0.170***	-0.195***	-0.127***	-0.007	-0.151***	-0.015
	(0.013)	(0.029)	(0.033)	(0.045)	(0.047)	(0.027)	(0.024)
Farm land area	0.000	0.006*	0.023	-0.001	-0.010	0.002	0.001
	(0.001)	(0.003)	(0.026)	(0.002)	(0.007)	(0.002)	(0.001)
Non-food cash crop	0.097***	0.210***	0.031	0.203	0.323***	0.010	-0.007
	(0.015)	(0.031)	(0.047)	(0.217)	(0.060)	(0.028)	(0.027)
Motorbike (time average)	0.071**	1.170***	0.313	0.110	-0.059	0.503***	0.222***
	(0.034)	(0.216)	(0.213)	(0.119)	(0.053)	(0.076)	(0.080)
Phone (time average)	0.547***	0.533***	0.458***	0.335***	0.821***	0.408***	0.398***
	(0.025)	(0.056)	(0.076)	(0.092)	(0.059)	(0.054)	(0.060)
Electricity (time average)	0.697***	0.666***	0.726***	0.509***	0.852***	0.549***	0.497***
	(0.027)	(0.067)	(0.110)	(0.124)	(0.057)	(0.055)	(0.064)
Wage employment (time average)	0.241***	0.500***	0.386***	0.186*	0.250***	0.118***	0.152***
	(0.025)	(0.081)	(0.079)	(0.104)	(0.064)	(0.042)	(0.053)
Nonfarm business (time average)	0.189***	0.085	0.340***	0.443***	-0.010	0.236***	0.357***
	(0.022)	(0.060)	(0.068)	(0.081)	(0.051)	(0.043)	(0.051)
Production diversity (time average)	-0.050***	0.004	-0.078***	-0.272***	-0.077***	-0.102***	-0.007
	(0.007)	(0.014)	(0.021)	(0.039)	(0.018)	(0.013)	(0.015)
Constant	4.465***	3.169***	5.319***	4.558***	4.524***	4.368***	3.964***
	(0.046)	(0.068)	(0.088)	(0.108)	(0.074)	(0.063)	(0.082)
Year and country dummies	yes	yes	yes	yes	yes	yes	yes
No. of obs.	89742	13511	9163	7046	18592	21117	20313
P-value	0.000	0.000	0.000	0.000	0.000	0.000	0.000

Table 3. Associations between farm-level production diversity and household dietary diversity by distance to the nearest urban center with more than 20,000 population

*** p<0.01, **p<0.05, * p<0.1, farm-level production diversity measured in terms of food groups. The p-values of coefficients are obtained from two-sided z-tests with standard errors clustered at the household level. The sample is smaller than the full sample because of missing data for the distance variable.

	Correlated random effects models						
	All	Ethiopia	Malawi	Niger	Nigeria	Tanzania	Uganda
Production diversity	0.061***	0.011	0.083**	0.138***	0.099***	0.090***	0.042*
	(0.008)	(0.013)	(0.035)	(0.038)	(0.016)	(0.017)	(0.025)
Distance	-0.005***	-0.004***	-0.010***	-0.008***	-0.002**	-0.005***	-0.014***
	(0.000)	(0.001)	(0.003)	(0.001)	(0.001)	(0.001)	(0.002)
Production diversity*distance	0.001***	0.001***	0.002***	0.002***	-0.000	0.000**	0.002**
	(0.000)	(0.000)	(0.001)	(0.000)	(0.000)	(0.000)	(0.001)
Household size	-0.003	0.048***	-0.011	0.016**	-0.025***	0.004	0.022***
	(0.003)	(0.007)	(0.013)	(0.006)	(0.004)	(0.006)	(0.007)
Share of dependent members	0.075***	-0.015	-0.401***	-0.023	0.139***	0.132*	0.114
	(0.029)	(0.054)	(0.120)	(0.097)	(0.049)	(0.071)	(0.093)
Head age	-0.000	-0.003***	-0.004**	0.002*	0.004***	-0.004***	-0.007***
	(0.000)	(0.001)	(0.002)	(0.001)	(0.001)	(0.001)	(0.001)
Head female	0.040**	-0.015	-0.150**	0.120**	0.217***	0.075**	-0.078*
	(0.017)	(0.033)	(0.066)	(0.057)	(0.035)	(0.038)	(0.047)
Head literacy	0.288***	0.396***	0.351***	0.318***	0.192***	0.290***	0.211***
	(0.015)	(0.030)	(0.065)	(0.044)	(0.027)	(0.039)	(0.047)
Motorbike	0.066**	-0.141	0.253	0.245**	0.037	0.122	0.064
	(0.030)	(0.132)	(0.384)	(0.096)	(0.038)	(0.085)	(0.087)
Phone	0.173***	0.188***	0.235**	0.098	0.186***	0.257***	0.147***
	(0.021)	(0.037)	(0.099)	(0.075)	(0.038)	(0.048)	(0.056)
Electricity	0.102***	0.142***	0.721***	0.214**	0.084*	0.138*	0.129
	(0.028)	(0.045)	(0.182)	(0.106)	(0.045)	(0.083)	(0.104)
Wage employment	0.106***	-0.037	0.169	0.217***	0.214***	0.113***	0.017
	(0.022)	(0.054)	(0.109)	(0.084)	(0.048)	(0.039)	(0.046)
Nonfarm business	0.189***	0.211***	0.248***	-0.174***	0.278***	0.198***	0.214***
	(0.020)	(0.047)	(0.078)	(0.065)	(0.039)	(0.041)	(0.053)
Weather shock	-0.055***	-0.159***	-0.164***	-0.086*	-0.005	-0.012	0.130***
	(0.016)	(0.029)	(0.053)	(0.045)	(0.047)	(0.040)	(0.036)
Farm land area	0.001	0.006	0.037	-0.001	-0.009	0.003	0.003**
	(0.001)	(0.004)	(0.042)	(0.002)	(0.007)	(0.002)	(0.001)
Non-food cash crop	0.118***	0.210***	-0.014	0.175	0.314***	-0.012	-0.006
	(0.019)	(0.031)	(0.073)	(0.216)	(0.060)	(0.038)	(0.040)
Motorbike (time average)	0.019	1.152***	-0.089	0.125	-0.053	0.465***	0.353***
	(0.040)	(0.215)	(0.479)	(0.119)	(0.053)	(0.110)	(0.128)
Phone (time average)	0.618***	0.533***	0.410***	0.212**	0.813***	0.585***	0.520***
	(0.029)	(0.056)	(0.123)	(0.093)	(0.059)	(0.068)	(0.081)
Electricity (time average)	0.775***	0.616***	0.326	0.376***	0.837***	0.521***	0.777***
	(0.036)	(0.068)	(0.207)	(0.126)	(0.058)	(0.099)	(0.133)
Wage employment (time average)	0.216***	0.481***	0.313**	0.072	0.248***	0.048	0.039
	(0.031)	(0.081)	(0.133)	(0.104)	(0.064)	(0.058)	(0.074)
Nonfarm business (time average)	0.136***	0.093	0.278***	0.315***	-0.010	0.195***	0.187**
	(0.027)	(0.060)	(0.106)	(0.081)	(0.051)	(0.058)	(0.074)
Production diversity (time average)	-0.025***	0.006	-0.045	-0.222***	-0.077***	-0.037**	0.010
	(0.008)	(0.014)	(0.035)	(0.039)	(0.018)	(0.018)	(0.026)
Constant	4.646***	3.329***	5.686***	5.061***	4.589***	4.700***	4.672***
	(0.057)	(0.073)	(0.145)	(0.118)	(0.079)	(0.092)	(0.125)
Year and country dummies	yes	yes	yes	yes	yes	yes	yes
No. of obs.	61916	13494	3543	6907	18590	11364	8018
P-value	0.000	0.000	0.000	0.000	0.000	0.000	0.000

Table 4. Associations between farm-level production diversity and household dietary diversity from own production

*** p<0.01, **p<0.05, * p<0.1, farm-level production diversity measured in terms of food groups. The p-values of coefficients are obtained from two-sided z-tests with standard errors clustered at the household level

	Correlated random effects models						
	All	Ethiopia	Malawi	Niger	Nigeria	Tanzania	Uganda
Production diversity	0.229***	0.073***	0.317***	0.197***	0.232***	0.295***	0.226***
	(0.005)	(0.011)	(0.013)	(0.022)	(0.012)	(0.012)	(0.010)
Household size	0.020***	0.030***	-0.003	-0.000	0.011***	0.027***	0.033***
	(0.002)	(0.005)	(0.007)	(0.004)	(0.003)	(0.004)	(0.005)
Share of dependent members	-0.032*	-0.020	-0.029	-0.014	-0.036	-0.044	-0.041
	(0.018)	(0.040)	(0.057)	(0.048)	(0.032)	(0.042)	(0.046)
Head age	0.002***	0.002***	0.003***	-0.001*	0.001	-0.002***	0.003***
	(0.000)	(0.001)	(0.001)	(0.001)	(0.001)	(0.001)	(0.001)
Head female	-0.037***	-0.066***	-0.068**	-0.129***	-0.043*	-0.062***	-0.013
	(0.011)	(0.023)	(0.033)	(0.026)	(0.023)	(0.023)	(0.026)
Head literacy	0.082***	0.093***	0.059	0.060**	-0.026	0.033	0.170***
	(0.011)	(0.024)	(0.036)	(0.024)	(0.019)	(0.026)	(0.026)
Motorbike	0.000	-0.147	0.229*	0.035	-0.053**	0.067	0.014
	(0.020)	(0.125)	(0.125)	(0.043)	(0.027)	(0.055)	(0.046)
Phone	0.015	0.024	0.009	-0.101**	0.054**	-0.051	0.110***
	(0.015)	(0.035)	(0.042)	(0.041)	(0.027)	(0.037)	(0.031)
Electricity	-0.029*	-0.153***	-0.062	0.105***	-0.005	-0.156***	0.069**
	(0.018)	(0.041)	(0.063)	(0.040)	(0.032)	(0.044)	(0.031)
Wage employment	-0.071***	-0.016	-0.085*	0.102**	0.003	-0.023	-0.169***
	(0.014)	(0.034)	(0.045)	(0.043)	(0.029)	(0.028)	(0.025)
Nonfarm business	0.007	-0.022	0.052	-0.003	0.022	0.016	-0.026
	(0.014)	(0.041)	(0.037)	(0.038)	(0.028)	(0.029)	(0.028)
Weather shock	-0.013	-0.141***	-0.103***	-0.003	0.105***	-0.079***	-0.022
	(0.011)	(0.026)	(0.028)	(0.028)	(0.036)	(0.023)	(0.023)
Farm land area	0.005***	0.020***	0.146***	0.001	0.016**	0.016***	0.004*
	(0.001)	(0.007)	(0.029)	(0.001)	(0.007)	(0.005)	(0.002)
Non-food cash crop	0.169***	-0.019	0.165***	0.371**	0.164***	0.219***	0.195***
	(0.014)	(0.028)	(0.043)	(0.147)	(0.046)	(0.029)	(0.025)
Motorbike (time average)	0.096***	0.187	-0.129	-0.075	0.223***	-0.001	0.069
	(0.026)	(0.171)	(0.178)	(0.053)	(0.037)	(0.066)	(0.067)
Phone (time average)	0.017	-0.080*	-0.034	-0.064	0.073*	-0.089*	0.016
	(0.021)	(0.048)	(0.060)	(0.051)	(0.039)	(0.048)	(0.052)
Electricity (time average)	-0.111***	-0.221***	-0.003	-0.237***	-0.299***	0.028	-0.109**
	(0.022)	(0.054)	(0.077)	(0.050)	(0.041)	(0.051)	(0.051)
Wage employment (time average)	-0.153***	-0.207***	-0.023	-0.173***	-0.198***	-0.202***	-0.180***
	(0.019)	(0.046)	(0.062)	(0.049)	(0.039)	(0.037)	(0.043)
Nonfarm business (time average)	-0.253***	-0.197***	-0.224***	-0.065	-0.189***	-0.264***	-0.347***
	(0.018)	(0.048)	(0.053)	(0.046)	(0.035)	(0.038)	(0.044)
Production diversity (time average)	0.300***	0.279***	0.272***	0.231***	0.212***	0.262***	0.459***
	(0.006)	(0.013)	(0.017)	(0.023)	(0.014)	(0.013)	(0.014)
Constant	-0.188***	0.472***	0.014	0.117**	0.773***	0.483***	0.053
	(0.034)	(0.049)	(0.066)	(0.056)	(0.046)	(0.054)	(0.064)
Year and country dummies	yes	yes	yes	yes	yes	yes	yes
No. of obs.	82550	10583	9155	7044	18429	17046	20293
P-value	0.000	0.000	0.000	0.000	0.000	0.000	0.000

Table 5. Association between farm-level production diversity and household dietary diversity from market purchases

*** p<0.01, **p<0.05, * p<0.1, farm-level production diversity measured in terms of food groups. The p-values of coefficients are obtained from two-sided z-tests with standard errors clustered at the household level

	Correlated random effects models						
	All	Ethiopia	Malawi	Niger	Nigeria	Tanzania	Uganda
Production diversity	0.004	0.029**	-0.012	0.228***	0.015	-0.001	-0.005
	(0.006)	(0.013)	(0.017)	(0.038)	(0.016)	(0.014)	(0.012)
Household size	0.001	0.034***	0.017*	0.017**	-0.009*	-0.010	0.010
	(0.003)	(0.008)	(0.010)	(0.007)	(0.005)	(0.006)	(0.006)
Share of dependent members	-0.036	-0.007	-0.406***	-0.078	0.076	-0.039	0.044
	(0.029)	(0.066)	(0.086)	(0.107)	(0.055)	(0.068)	(0.064)
Head age	-0.004***	-0.005***	-0.007***	0.001	0.001	-0.002*	-0.007***
	(0.001)	(0.001)	(0.002)	(0.002)	(0.001)	(0.001)	(0.001)
Head female	0.017	-0.008	-0.129**	0.088	0.214***	0.059	0.052
	(0.018)	(0.039)	(0.051)	(0.064)	(0.040)	(0.037)	(0.038)
Head literacy	0.295***	0.338***	0.304***	0.364***	0.266***	0.430***	0.226***
	(0.016)	(0.036)	(0.052)	(0.048)	(0.030)	(0.038)	(0.035)
Motorbike	0.124***	0.053	0.218	0.233**	0.051	0.078	0.264***
	(0.029)	(0.172)	(0.146)	(0.100)	(0.042)	(0.078)	(0.058)
Phone	0.265***	0.205***	0.430***	0.148*	0.224***	0.401***	0.284***
	(0.020)	(0.045)	(0.060)	(0.081)	(0.042)	(0.047)	(0.038)
Electricity	0.266***	0.405***	0.530***	0.225**	0.134***	0.099	0.459***
	(0.024)	(0.054)	(0.096)	(0.109)	(0.050)	(0.062)	(0.039)
Wage employment	0.215***	-0.062	0.318***	0.284***	0.228***	0.205***	0.198***
	(0.019)	(0.065)	(0.065)	(0.089)	(0.053)	(0.037)	(0.031)
Nonfarm business	0.290***	0.301***	0.367***	-0.174**	0.339***	0.319***	0.278***
	(0.019)	(0.061)	(0.049)	(0.072)	(0.044)	(0.041)	(0.034)
Weather shock	-0.050***	-0.129***	-0.094**	-0.147***	-0.096*	-0.068**	0.025
	(0.015)	(0.035)	(0.039)	(0.050)	(0.053)	(0.034)	(0.027)
Farm land area	-0.001	-0.013**	-0.010	-0.001	-0.019	-0.006	0.000
	(0.001)	(0.006)	(0.029)	(0.002)	(0.011)	(0.004)	(0.002)
Non-food cash crop	-0.037**	0.234***	-0.005	0.070	0.207***	-0.109***	-0.124***
	(0.018)	(0.037)	(0.057)	(0.243)	(0.066)	(0.037)	(0.033)
Motorbike (time average)	0.041	0.697***	0.460*	0.146	-0.122**	0.553***	0.182*
	(0.040)	(0.257)	(0.238)	(0.125)	(0.058)	(0.101)	(0.096)
Phone (time average)	0.665***	0.667***	0.540***	0.467***	0.779***	0.692***	0.507***
	(0.030)	(0.067)	(0.092)	(0.102)	(0.064)	(0.067)	(0.070)
Electricity (time average)	0.815***	0.859***	0.905***	0.671***	1.043***	0.809***	0.496***
	(0.033)	(0.080)	(0.127)	(0.130)	(0.064)	(0.077)	(0.076)
Wage employment (time average)	0.351***	0.788***	0.549***	0.277**	0.370***	0.200***	0.418***
	(0.029)	(0.094)	(0.094)	(0.112)	(0.070)	(0.054)	(0.063)
Nonfarm business (time average)	0.458***	0.349***	0.675***	0.598***	0.134**	0.505***	0.772***
	(0.027)	(0.075)	(0.082)	(0.089)	(0.057)	(0.057)	(0.061)
Production diversity (time average)	-0.248***	-0.202***	-0.247***	-0.329***	-0.172***	-0.310***	-0.274***
	(0.008)	(0.017)	(0.025)	(0.043)	(0.021)	(0.017)	(0.018)
Constant	4.462***	2.305***	4.477***	4.218***	3.745***	3.645***	3.312***
	(0.053)	(0.085)	(0.109)	(0.118)	(0.082)	(0.085)	(0.097)
Year and country dummies	yes	yes	yes	yes	yes	yes	yes
No. of obs.	82550	10583	9155	7044	18429	17046	20293
P-value	0.000	0.000	0.000	0.000	0.000	0.000	0.000

Table 6. Association between farm-level production diversity and household dietary diversity (using the same sample for estimating village-, town-, and district-level production diversity)

*** p<0.01, **p<0.05, * p<0.1, farm-level production diversity measured in terms of food groups. The p-values of coefficients are obtained from two-sided z-tests with standard errors clustered at the household level

	Correlated random effects models						
	All	Ethiopia	Malawi	Niger	Nigeria	Tanzania	Uganda
Farm-level production diversity	0.105***	0.054***	0.124***	0.242***	0.080***	0.193***	0.099***
	(0.006)	(0.011)	(0.019)	(0.035)	(0.016)	(0.014)	(0.011)
Household size	-0.008***	0.031***	-0.010	0.013**	-0.029***	-0.009*	0.012**
	(0.002)	(0.007)	(0.011)	(0.007)	(0.005)	(0.005)	(0.006)
Share of dependent members	0.047*	-0.047	-0.268***	-0.133	0.156***	0.048	0.064
	(0.028)	(0.057)	(0.103)	(0.100)	(0.054)	(0.065)	(0.059)
Head age	0.000	-0.004***	-0.002	0.003**	0.005***	-0.002**	-0.001
	(0.000)	(0.001)	(0.002)	(0.001)	(0.001)	(0.001)	(0.001)
Head female	0.070***	-0.003	-0.113*	0.088	0.315***	0.049	0.059*
	(0.017)	(0.036)	(0.060)	(0.060)	(0.039)	(0.037)	(0.034)
Head literacy	0.305***	0.385***	0.296***	0.348***	0.191***	0.275***	0.327***
	(0.015)	(0.031)	(0.058)	(0.046)	(0.029)	(0.035)	(0.034)
Motorbike	0.120***	-0.227	0.278	0.301***	0.016	0.253***	0.230***
	(0.029)	(0.158)	(0.191)	(0.111)	(0.042)	(0.085)	(0.056)
Phone	0.169***	0.181***	0.097	0.089	0.163***	0.244***	0.245***
	(0.019)	(0.038)	(0.069)	(0.078)	(0.041)	(0.048)	(0.037)
Electricity	0.151***	0.156***	0.211	0.302**	0.083*	0.051	0.342***
	(0.023)	(0.046)	(0.139)	(0.123)	(0.050)	(0.055)	(0.038)
Wage employment	0.127***	0.087	0.145*	0.199**	0.257***	0.107***	0.042
	(0.020)	(0.063)	(0.081)	(0.090)	(0.057)	(0.038)	(0.030)
Nonfarm business	0.208***	0.227***	0.240***	-0.175**	0.308***	0.211***	0.196***
	(0.018)	(0.050)	(0.056)	(0.068)	(0.043)	(0.040)	(0.032)
Weather shock	-0.069***	-0.146***	-0.166***	-0.132***	0.033	-0.173***	-0.015
	(0.014)	(0.029)	(0.044)	(0.045)	(0.047)	(0.034)	(0.025)
Farm land area	-0.001	0.008**	0.034	-0.001	-0.009	-0.002	0.000
	(0.001)	(0.004)	(0.032)	(0.002)	(0.007)	(0.003)	(0.002)
Non-food cash crop	0.121***	0.212***	0.036	0.208	0.334***	0.020	0.017
	(0.016)	(0.031)	(0.061)	(0.216)	(0.061)	(0.033)	(0.028)
Motorbike (time average)	0.042	1.059***	-0.002	0.053	0.065	0.281**	0.144*
	(0.040)	(0.266)	(0.324)	(0.133)	(0.057)	(0.113)	(0.087)
Phone (time average)	0.570***	0.471***	0.748***	0.334***	0.772***	0.443***	0.374***
	(0.028)	(0.057)	(0.108)	(0.095)	(0.060)	(0.066)	(0.063)
Electricity (time average)	0.701***	0.515***	1.010***	0.408***	0.843***	0.602***	0.430***
	(0.031)	(0.068)	(0.176)	(0.140)	(0.062)	(0.074)	(0.070)
Wage employment (time average)	0.202***	0.498***	0.404***	0.216*	0.140*	0.045	0.179***
	(0.029)	(0.098)	(0.115)	(0.111)	(0.074)	(0.055)	(0.056)
Nonfarm business (time average)	0.170***	0.098	0.327***	0.454***	-0.053	0.302***	0.343***
	(0.026)	(0.064)	(0.099)	(0.084)	(0.056)	(0.057)	(0.054)
Production diversity (time average)	-0.022***	0.049***	-0.027	-0.257***	-0.034*	-0.120***	0.024
	(0.008)	(0.015)	(0.029)	(0.040)	(0.020)	(0.017)	(0.017)
Constant	4.442***	3.138***	4.889***	4.580***	4.475***	4.486***	3.866***
	(0.053)	(0.071)	(0.144)	(0.111)	(0.080)	(0.083)	(0.088)
Year and country dummies	yes	yes	yes	yes	yes	yes	yes
No. of obs.	65579	11410	4626	6536	15063	11830	16114
P-value	0.000	0.000	0.000	0.000	0.000	0.000	0.000

Table 7. Association between village-level production diversity and household dietary diversity

*** p<0.01, **p<0.05, * p<0.1, village-level production diversity measured in terms of food groups. The p-values of coefficients are obtained from two-sided z-tests with standard errors clustered at the household level

	Correlated random effects models						
	All	Ethiopia	Malawi	Niger	Nigeria	Tanzania	Uganda
Village-level production diversity	0.044***	0.052***	0.066**	0.112***	0.067***	0.055***	0.020
	(0.007)	(0.015)	(0.026)	(0.031)	(0.016)	(0.019)	(0.013)
No. of farmers in village	-0.049***	-0.049***	-0.038***	-0.063***	-0.092***	-0.048***	-0.031***
	(0.002)	(0.008)	(0.010)	(0.006)	(0.007)	(0.007)	(0.005)
Household size	0.004	0.052***	-0.000	0.019***	-0.015***	-0.002	0.026***
	(0.002)	(0.007)	(0.011)	(0.006)	(0.005)	(0.005)	(0.005)
Share of dependent members	0.046*	-0.054	-0.244**	-0.096	0.148***	0.053	0.053
	(0.028)	(0.057)	(0.103)	(0.100)	(0.054)	(0.066)	(0.060)
Head age	0.000	-0.003***	-0.002	0.001	0.003***	-0.001	-0.001
	(0.000)	(0.001)	(0.002)	(0.001)	(0.001)	(0.001)	(0.001)
Head female	0.035**	-0.052	-0.103*	0.045	0.256***	0.022	0.047
	(0.017)	(0.036)	(0.060)	(0.059)	(0.039)	(0.037)	(0.034)
Head literacy	0.298***	0.375***	0.307***	0.335***	0.174***	0.274***	0.324***
	(0.015)	(0.031)	(0.058)	(0.045)	(0.028)	(0.035)	(0.034)
Motorbike	0.124***	-0.213	0.247	0.326***	0.020	0.273***	0.235***
	(0.029)	(0.157)	(0.192)	(0.112)	(0.042)	(0.086)	(0.056)
Phone	0.172***	0.184***	0.101	0.103	0.164***	0.274***	0.250***
	(0.019)	(0.038)	(0.070)	(0.079)	(0.041)	(0.049)	(0.037)
Electricity	0.143***	0.163***	0.189	0.228*	0.075	0.045	0.337***
	(0.023)	(0.046)	(0.141)	(0.126)	(0.050)	(0.056)	(0.038)
Wage employment	0.127***	0.076	0.132	0.211**	0.240***	0.126***	0.037
	(0.020)	(0.063)	(0.081)	(0.091)	(0.057)	(0.039)	(0.031)
Nonfarm business	0.215***	0.224***	0.251***	-0.161**	0.299***	0.231***	0.205***
	(0.018)	(0.050)	(0.056)	(0.070)	(0.043)	(0.041)	(0.032)
Weather shock	-0.035**	-0.110***	-0.134***	-0.068	0.069	-0.143***	0.009
	(0.014)	(0.030)	(0.044)	(0.045)	(0.047)	(0.034)	(0.025)
Farm land area	0.001	0.014***	0.113***	-0.001	-0.004	0.003	0.002
	(0.001)	(0.004)	(0.036)	(0.002)	(0.005)	(0.003)	(0.002)
Non-food cash crop	0.164***	0.253***	0.096	0.300	0.333***	0.122***	0.051*
	(0.016)	(0.031)	(0.062)	(0.219)	(0.060)	(0.032)	(0.028)
Motorbike (time average)	0.044	1.025***	0.144	-0.001	0.078	0.269**	0.140
	(0.039)	(0.262)	(0.335)	(0.134)	(0.056)	(0.113)	(0.087)
Phone (time average)	0.527***	0.476***	0.700***	0.141	0.685***	0.408***	0.347***
	(0.028)	(0.057)	(0.109)	(0.097)	(0.060)	(0.066)	(0.062)
Electricity (time average)	0.610***	0.373***	0.803***	0.201	0.707***	0.555***	0.374***
	(0.031)	(0.069)	(0.178)	(0.145)	(0.062)	(0.074)	(0.069)
Wage employment (time average)	0.122***	0.426***	0.264**	0.058	0.103	-0.011	0.112**
	(0.029)	(0.097)	(0.117)	(0.111)	(0.074)	(0.056)	(0.055)
Nonfarm business (time average)	0.124***	0.057	0.236**	0.364***	-0.077	0.245***	0.304***
	(0.026)	(0.063)	(0.100)	(0.085)	(0.055)	(0.057)	(0.054)
Production diversity (time average)	0.084***	0.079***	-0.046	-0.048	0.136***	-0.020	0.185***
	(0.009)	(0.018)	(0.040)	(0.035)	(0.020)	(0.023)	(0.022)
Constant	4.697***	3.066***	5.391***	5.305***	4.569***	4.691***	3.278***
	(0.061)	(0.103)	(0.215)	(0.136)	(0.102)	(0.108)	(0.127)
Year and country dummies	yes	yes	yes	yes	yes	yes	yes
No. of obs.	65579	11410	4626	6536	15063	11830	16114
P-value	0.000	0.000	0.000	0.000	0.000	0.000	0.000

Table 8. Association between town-level production diversity and household dietary diversity

*** p<0.01, **p<0.05, * p<0.1, town-level production diversity measured in terms of food groups. The p-values of coefficients are obtained from two-sided z-tests with standard errors clustered at the household level

	Correlated random effects models						
	All	Ethiopia	Malawi	Niger	Nigeria	Tanzania	Uganda
Town-level production diversity	0.025***	0.054***	0.035	0.080***	0.067***	-0.000	0.015
	(0.007)	(0.016)	(0.026)	(0.030)	(0.015)	(0.021)	(0.014)
No. of farmers in town	-0.002***	-0.006***	-0.013***	-0.001***	-0.019***	-0.014***	-0.017***
	(0.000)	(0.001)	(0.004)	(0.000)	(0.003)	(0.005)	(0.003)
Household size	0.002	0.050***	-0.001	0.011*	-0.020***	-0.001	0.026***
	(0.002)	(0.007)	(0.011)	(0.006)	(0.005)	(0.005)	(0.005)
Share of dependent members	0.035	-0.062	-0.256**	-0.145	0.156***	0.052	0.050
	(0.028)	(0.057)	(0.103)	(0.099)	(0.054)	(0.066)	(0.060)
Head age	0.001	-0.003***	-0.001	0.003**	0.004***	-0.001	-0.001
	(0.000)	(0.001)	(0.002)	(0.001)	(0.001)	(0.001)	(0.001)
Head female	0.048***	-0.053	-0.091	0.096	0.285***	0.021	0.046
	(0.017)	(0.036)	(0.060)	(0.059)	(0.039)	(0.037)	(0.034)
Head literacy	0.306***	0.369***	0.313***	0.354***	0.181***	0.279***	0.323***
	(0.015)	(0.031)	(0.059)	(0.046)	(0.028)	(0.035)	(0.034)
Motorbike	0.127***	-0.211	0.261	0.339***	0.016	0.262***	0.237***
	(0.029)	(0.157)	(0.192)	(0.112)	(0.042)	(0.086)	(0.056)
Phone	0.177***	0.181***	0.106	0.115	0.167***	0.272***	0.252***
	(0.019)	(0.038)	(0.070)	(0.079)	(0.041)	(0.049)	(0.037)
Electricity	0.150***	0.163***	0.196	0.264**	0.075	0.022	0.342***
	(0.023)	(0.046)	(0.140)	(0.125)	(0.049)	(0.056)	(0.038)
Wage employment	0.128***	0.078	0.134*	0.210**	0.246***	0.132***	0.036
	(0.020)	(0.063)	(0.081)	(0.091)	(0.057)	(0.039)	(0.031)
Nonfarm business	0.218***	0.221***	0.253***	-0.161**	0.306***	0.235***	0.203***
	(0.018)	(0.050)	(0.056)	(0.070)	(0.043)	(0.041)	(0.032)
Weather shock	-0.053***	-0.116***	-0.146***	-0.142***	0.049	-0.154***	0.006
	(0.014)	(0.030)	(0.043)	(0.045)	(0.047)	(0.034)	(0.025)
Farm land area	0.000	0.012***	0.099***	-0.002	-0.005	0.004	0.002
	(0.001)	(0.004)	(0.035)	(0.002)	(0.006)	(0.003)	(0.002)
Non-food cash crop	0.156***	0.268***	0.093	0.215	0.336***	0.135***	0.050*
	(0.016)	(0.031)	(0.062)	(0.216)	(0.060)	(0.033)	(0.028)
Motorbike (time average)	0.029	0.955***	0.077	0.009	0.048	0.280**	0.140
	(0.040)	(0.261)	(0.327)	(0.134)	(0.057)	(0.114)	(0.087)
Phone (time average)	0.554***	0.463***	0.710***	0.290***	0.735***	0.400***	0.346***
	(0.028)	(0.057)	(0.109)	(0.096)	(0.060)	(0.067)	(0.062)
Electricity (time average)	0.685***	0.518***	0.837***	0.511***	0.814***	0.557***	0.375***
	(0.031)	(0.067)	(0.177)	(0.141)	(0.061)	(0.075)	(0.069)
Wage employment (time average)	0.170***	0.450***	0.330***	0.179	0.158**	-0.006	0.116**
	(0.029)	(0.097)	(0.115)	(0.111)	(0.074)	(0.056)	(0.055)
Nonfarm business (time average)	0.148***	0.101	0.270***	0.404***	-0.047	0.258***	0.311***
	(0.026)	(0.063)	(0.100)	(0.085)	(0.055)	(0.057)	(0.054)
Production diversity (time average)	0.083***	0.049**	-0.037	0.048	0.116***	0.012	0.190***
	(0.009)	(0.019)	(0.039)	(0.037)	(0.019)	(0.025)	(0.023)
Constant	4.114***	2.810***	5.262***	3.948***	3.977***	4.668***	3.142***
	(0.062)	(0.094)	(0.222)	(0.147)	(0.093)	(0.115)	(0.131)
Year and country dummies	yes	yes	yes	yes	yes	yes	yes
No. of obs.	65579	11410	4626	6536	15063	11830	16114
P-value	0.000	0.000	0.000	0.000	0.000	0.000	0.000

Table 9. Association between district-level production diversity and household dietary diversity

*** p<0.01, **p<0.05, * p<0.1, district-level production diversity measured in terms of food groups. The p-values of coefficients are obtained from two-sided z-tests with standard errors clustered at the household level

	Correlated random effects models						
	All	Ethiopia	Malawi	Niger	Nigeria	Tanzania	Uganda
District-level production diversity	0.028***	0.058***	-0.043	0.028	0.111***	-0.035	0.015
	(0.009)	(0.016)	(0.042)	(0.044)	(0.020)	(0.026)	(0.017)
No. of farmers in district	-0.001***	-0.005***	-0.004***	0.000*	-0.004***	0.001	0.000
	(0.000)	(0.001)	(0.001)	(0.000)	(0.000)	(0.001)	(0.001)
Household size	0.002	0.051***	-0.002	0.014**	-0.022***	-0.001	0.030***
	(0.002)	(0.007)	(0.011)	(0.006)	(0.005)	(0.005)	(0.005)
Share of dependent members	0.039	-0.050	-0.269***	-0.131	0.137**	0.051	0.057
	(0.028)	(0.057)	(0.103)	(0.100)	(0.054)	(0.066)	(0.060)
Head age	0.001*	-0.004***	-0.002	0.003**	0.005***	-0.001	-0.001
	(0.000)	(0.001)	(0.002)	(0.001)	(0.001)	(0.001)	(0.001)
Head female	0.049***	-0.057	-0.106*	0.080	0.306***	0.026	0.035
	(0.017)	(0.036)	(0.060)	(0.059)	(0.039)	(0.037)	(0.034)
Head literacy	0.308***	0.371***	0.300***	0.360***	0.200***	0.278***	0.318***
	(0.015)	(0.031)	(0.059)	(0.046)	(0.029)	(0.035)	(0.034)
Motorbike	0.126***	-0.214	0.264	0.339***	0.015	0.256***	0.236***
	(0.029)	(0.158)	(0.190)	(0.112)	(0.042)	(0.086)	(0.056)
Phone	0.176***	0.181***	0.112	0.118	0.162***	0.273***	0.249***
	(0.019)	(0.038)	(0.070)	(0.079)	(0.041)	(0.049)	(0.037)
Electricity	0.148***	0.163***	0.221	0.260**	0.084*	0.033	0.341***
	(0.023)	(0.046)	(0.140)	(0.125)	(0.049)	(0.056)	(0.038)
Wage employment	0.129***	0.077	0.138*	0.221**	0.245***	0.130***	0.036
	(0.020)	(0.063)	(0.081)	(0.091)	(0.057)	(0.039)	(0.031)
Nonfarm business	0.217***	0.220***	0.258***	-0.157**	0.302***	0.235***	0.202***
	(0.018)	(0.050)	(0.056)	(0.070)	(0.043)	(0.041)	(0.032)
Weather shock	-0.050***	-0.122***	-0.163***	-0.115**	0.030	-0.155***	0.024
	(0.014)	(0.030)	(0.043)	(0.045)	(0.047)	(0.034)	(0.025)
Farm land area	0.001	0.013***	0.092***	-0.001	-0.005	0.003	0.002
	(0.001)	(0.004)	(0.034)	(0.002)	(0.006)	(0.003)	(0.002)
Non-food cash crop	0.176***	0.296***	0.091	0.214	0.294***	0.121***	0.065**
	(0.016)	(0.031)	(0.062)	(0.217)	(0.060)	(0.032)	(0.028)
Motorbike (time average)	0.048	0.984***	0.004	-0.008	0.095*	0.279**	0.126
	(0.040)	(0.262)	(0.314)	(0.134)	(0.057)	(0.114)	(0.087)
Phone (time average)	0.553***	0.458***	0.718***	0.301***	0.744***	0.410***	0.336***
	(0.028)	(0.057)	(0.108)	(0.096)	(0.060)	(0.066)	(0.063)
Electricity (time average)	0.653***	0.472***	0.866***	0.457***	0.803***	0.574***	0.315***
	(0.031)	(0.067)	(0.174)	(0.141)	(0.061)	(0.074)	(0.069)
Wage employment (time average)	0.154***	0.426***	0.351***	0.180	0.106	-0.006	0.073
	(0.029)	(0.097)	(0.114)	(0.111)	(0.074)	(0.056)	(0.056)
Nonfarm business (time average)	0.131***	0.095	0.276***	0.409***	-0.088	0.252***	0.291***
	(0.026)	(0.064)	(0.100)	(0.086)	(0.055)	(0.057)	(0.054)
Production diversity (time average)	0.069***	0.021	-0.019	0.060	-0.049*	0.087***	0.211***
	(0.012)	(0.020)	(0.066)	(0.053)	(0.027)	(0.034)	(0.030)
Constant	4.118***	2.955***	5.714***	3.916***	4.519***	4.215***	2.616***
	(0.072)	(0.096)	(0.375)	(0.194)	(0.169)	(0.168)	(0.182)
Year and country dummies	yes	yes	yes	yes	yes	yes	yes
No. of obs.	65579	11410	4626	6536	15063	11830	16114
P-value	0.000	0.000	0.000	0.000	0.000	0.000	0.000

Table 10. Number of observations by survey and country

*The surveyed year indicate the starting time of the survey. The time duration of surveys by countries are as follows: In Ethiopia, 3 waves (09/11-03/12, 09/13-04/14, 09/15-04/16), in Malawi, 4 waves (03/10-03/11, 04/13-11/13, 04/16-04/17, 04/19-03/20), in Niger, 2 waves (07/11-01/12, 09/14-03/15), in Nigeria, 4 waves (08/10-04/11; 09/12-04/13, 08/15-04/16; 07/18-02/2019), in Tanzania, 5 waves (10/08-09/09, 10/10-11/11, 10/12-11/13, 10/14-10/15, 01/19-01/22), and in Uganda, 7 waves (09/09-08/10, 11/10-10/11, 11/11-12/12, 09/13-08/14, 03/15-03/16, 03/18-02/19, 03/19-02/20).

Surveyed year*	All	Ethiopia	Malawi	Niger	Nigeria	Tanzania	Uganda
2008	3,176					3,176	
2009	2,837						2,837
2010	12,719		1,581		4,801	3,767	2,570
2011	10,467	3,786		3,930			2,751
2012	9,429				4,724	4,705	
2013	10,027	5,037	1,962				3,028
2014	7,298			3,116		4,182	
2015	12,323	4,688			4,504		3,131
2016	2,447		2,447				
2018	7,615				4,563		3,052
2019	11,404		3,173			5,287	2,944
Total observations	89,742	13,511	9,163	7,046	18,592	21,117	20,313

Table 11. Association between farm-level production diversity (species count)) and household dietary diversity (with different samples)

*** p<0.01, **p<0.05, * p<0.1. The p-values of coefficients are obtained from two-sided z-tests with standard errors clustered at the household level

	Correlated random effects models						
	All	Ethiopia	Malawi	Niger	Nigeria	Tanzania	Uganda
**(A) Farmer sample**							
	0.049***	0.017***	0.095***	0.156***	0.054***	0.066***	0.054***
	(0.003)	(0.006)	(0.011)	(0.019)	(0.010)	(0.006)	(0.006)
No. of obs.	67221	9981	7359	5312	12882	14921	16766
**(B) Balanced sample**							
	0.036***	0.019***	0.091***	0.141***	0.037***	0.036**	0.050***
	(0.004)	(0.006)	(0.011)	(0.018)	(0.013)	(0.015)	(0.008)
No. of obs.	35207	9447	5604	5954	4812	1585	7805
**(C) Whole sample**							
	0.044***	0.017***	0.090***	0.140***	0.047***	0.058***	0.048***
	(0.003)	(0.006)	(0.010)	(0.018)	(0.008)	(0.006)	(0.005)
No. of obs.	89742	13511	9163	7046	18592	21117	20313
**(D) Farmer sample with Heckman sample selection correction**							
	0.049***	0.018***	0.094***	0.156***	0.054***	0.067***	0.056***
	(0.003)	(0.006)	(0.011)	(0.019)	(0.010)	(0.006)	(0.006)
No. of obs.	67221	9981	7359	5312	12882	14921	16766
**(E) Balanced sample with Heckman sample selection correction**							
	0.028***	0.021***	0.112***	0.126***	-0.015	-0.004	0.021**
	(0.004)	(0.006)	(0.012)	(0.019)	(0.020)	(0.020)	(0.011)
No. of obs.	35207	9447	5604	5954	4812	1585	7805

Table 12. Association between farm-level production diversity (food groups) and household dietary diversity (with different samples)

*** p<0.01, **p<0.05, * p<0.1. The p-values of coefficients are obtained from two-sided z-tests with standard errors clustered at the household level

	Correlated random effects models						
	All	Ethiopia	Malawi	Niger	Nigeria	Tanzania	Uganda
**(A) Farmer sample**							
	0.120***	0.059***	0.127***	0.286***	0.121***	0.181***	0.114***
	(0.006)	(0.012)	(0.017)	(0.040)	(0.018)	(0.013)	(0.012)
No. of obs.	67221	9981	7359	5312	12882	14921	16766
**(B) Balanced sample**							
	0.082***	0.054***	0.119***	0.246***	0.077***	0.165***	0.093***
	(0.007)	(0.011)	(0.016)	(0.035)	(0.023)	(0.030)	(0.014)
No. of obs.	35207	9447	5604	5954	4812	1585	7805
**(C) Whole sample**							
	0.100***	0.051***	0.116***	0.245***	0.095***	0.144***	0.097***
	(0.005)	(0.011)	(0.014)	(0.035)	(0.014)	(0.011)	(0.010)
No. of obs.	89742	13511	9163	7046	18592	21117	20313
**(D) Farmer sample with Heckman sample selection correction**							
	0.120***	0.061***	0.125***	0.286***	0.121***	0.183***	0.115***
	(0.006)	(0.012)	(0.017)	(0.040)	(0.018)	(0.013)	(0.012)
No. of obs.	67221	9981	7359	5312	12882	14921	16766
**(E) Balanced sample with Heckman sample selection correction**							
	0.072***	0.093***	0.166***	0.210***	0.312*	0.676	-0.153**
	(0.008)	(0.012)	(0.019)	(0.038)	(0.179)	(0.482)	(0.072)
No. of obs.	35207	9447	5604	5954	4812	1585	7805

Table 13. Associations between farm-level production diversity and household dietary diversity from own production (with different samples)

*** p<0.01, **p<0.05, * p<0.1. The p-values of coefficients are obtained from two-sided z-tests with standard errors clustered at the household level

	Correlated random effects models						
	All	Ethiopia	Malawi	Niger	Nigeria	Tanzania	Uganda
**(A) Farmer sample**							
	0.200***	0.069***	0.279***	0.230***	0.182***	0.304***	0.187***
	(0.006)	(0.012)	(0.015)	(0.027)	(0.016)	(0.015)	(0.012)
No. of obs.	62289	7874	7351	5310	12811	12194	16749
**(B) Balanced sample**							
	0.204***	0.075***	0.311***	0.198***	0.217***	0.344***	0.215***
	(0.007)	(0.012)	(0.016)	(0.022)	(0.020)	(0.031)	(0.015)
No. of obs.	33152	7449	5598	5952	4770	1582	7801
**(C) Whole sample**							
	0.229***	0.073***	0.317***	0.197***	0.232***	0.295***	0.226***
	(0.005)	(0.011)	(0.013)	(0.022)	(0.012)	(0.012)	(0.010)
No. of obs.	82550	10583	9155	7044	18429	17046	20293
**(D) Farmer sample with Heckman sample selection correction**							
	0.200***	0.067***	0.275***	0.230***	0.182***	0.305***	0.188***
	(0.006)	(0.012)	(0.015)	(0.027)	(0.016)	(0.015)	(0.012)
No. of obs.	62289	7874	7351	5310	12811	12194	16749
**(E) Balanced sample with Heckman sample selection correction**							
	0.151***	0.074***	0.334***	0.100***	-0.035	-0.022	-0.028
	(0.008)	(0.013)	(0.018)	(0.023)	(0.121)	(0.223)	(0.060)
No. of obs.	33152	7449	5598	5952	4770	1582	7801

Table 14. Associations between farm-level production diversity and household dietary diversity from market purchases (with different samples)

*** p<0.01, **p<0.05, * p<0.1. The p-values of coefficients are obtained from two-sided z-tests with standard errors clustered at the household level

	Correlated random effects models						
	All	Ethiopia	Malawi	Niger	Nigeria	Tanzania	Uganda
**(A) Farmer sample**							
	0.025***	0.031**	0.002	0.264***	0.036*	0.019	0.019
	(0.007)	(0.014)	(0.019)	(0.044)	(0.020)	(0.017)	(0.014)
No. of obs.	62289	7874	7351	5310	12811	12194	16749
**(B) Balanced sample**							
	0.001	0.029**	-0.011	0.229***	0.007	0.018	-0.019
	(0.009)	(0.013)	(0.020)	(0.038)	(0.026)	(0.039)	(0.018)
No. of obs.	33152	7449	5598	5952	4770	1582	7801
**(C) Whole sample**							
	0.004	0.029**	-0.012	0.228***	0.015	-0.001	-0.005
	(0.006)	(0.013)	(0.017)	(0.038)	(0.016)	(0.014)	(0.012)
No. of obs.	82550	10583	9155	7044	18429	17046	20293
**(D) Farmer sample with Heckman sample selection correction**							
	0.025***	0.032**	0.001	0.264***	0.036*	0.021	0.019
	(0.007)	(0.014)	(0.019)	(0.044)	(0.020)	(0.017)	(0.014)
No. of obs.	62289	7874	7351	5310	12811	12194	16749
**(E) Balanced sample with Heckman sample selection correction**							
	0.017*	0.091***	0.001	0.236***	0.286	-0.155	-0.376***
	(0.010)	(0.014)	(0.022)	(0.042)	(0.212)	(0.462)	(0.083)
No. of obs.	33152	7449	5598	5952	4770	1582	7801

Table 15. Associations between farm-level production diversity and household dietary diversity (using the same sample for estimating village-, town-, and district-level production diversity) (with different samples)

*** p<0.01, **p<0.05, * p<0.1. The p-values of coefficients are obtained from two-sided z-tests with standard errors clustered at the household level

	Correlated random effects models						
	All	Ethiopia	Malawi	Niger	Nigeria	Tanzania	Uganda
**(A) Farmer sample**							
	0.119***	0.059***	0.118***	0.285***	0.119***	0.206***	0.113***
	(0.007)	(0.012)	(0.021)	(0.040)	(0.018)	(0.015)	(0.013)
No. of obs.	57175	9949	4125	5278	12681	10533	14609
**(B) Balanced sample**							
	0.082***	0.056***	0.121***	0.244***	0.055**	0.193***	0.103***
	(0.008)	(0.011)	(0.020)	(0.035)	(0.026)	(0.033)	(0.016)
No. of obs.	30867	9299	4301	5552	4014	1113	6588
**(C) Whole sample**							
	0.105***	0.054***	0.124***	0.242***	0.080***	0.193***	0.099***
	(0.006)	(0.011)	(0.019)	(0.035)	(0.016)	(0.014)	(0.011)
No. of obs.	65579	11410	4626	6536	15063	11830	16114
**(D) Farmer sample with Heckman sample selection correction**							
	0.119***	0.061***	0.117***	0.284***	0.119***	0.206***	0.114***
	(0.007)	(0.012)	(0.021)	(0.040)	(0.018)	(0.015)	(0.013)
No. of obs.	57175	9949	4125	5278	12681	10533	14609
**(E) Balanced sample with Heckman sample selection correction**							
	0.060***	0.101***	0.158***	0.205***	0.499**	0.056	-0.212***
	(0.009)	(0.013)	(0.022)	(0.039)	(0.201)	(0.430)	(0.078)
No. of obs.	30867	9299	4301	5552	4014	1113	6588

Table 16. Associations between village-level production diversity and household dietary diversity (with different samples)

*** p<0.01, **p<0.05, * p<0.1. The p-values of coefficients are obtained from two-sided z-tests with standard errors clustered at the household level

	Correlated random effects models						
	All	Ethiopia	Malawi	Niger	Nigeria	Tanzania	Uganda
**(A) Farmer sample**							
	0.049***	0.051***	0.054*	0.165***	0.059***	0.070***	0.028**
	(0.008)	(0.016)	(0.028)	(0.036)	(0.018)	(0.020)	(0.014)
No. of obs.	57175	9949	4125	5278	12681	10533	14609
**(B) Balanced sample**							
	0.050***	0.052***	0.070***	0.111***	0.106***	0.068	0.028
	(0.010)	(0.016)	(0.026)	(0.031)	(0.026)	(0.048)	(0.019)
No. of obs.	30867	9299	4301	5552	4014	1113	6588
**(C) Whole sample**							
	0.044***	0.052***	0.066**	0.112***	0.067***	0.055***	0.020
	(0.007)	(0.015)	(0.026)	(0.031)	(0.016)	(0.019)	(0.013)
No. of obs.	65579	11410	4626	6536	15063	11830	16114
**(D) Farmer sample with Heckman sample selection correction**							
	0.049***	0.051***	0.050*	0.165***	0.060***	0.067***	0.029**
	(0.008)	(0.016)	(0.028)	(0.036)	(0.018)	(0.020)	(0.014)
No. of obs.	57175	9949	4125	5278	12681	10533	14609
**(E) Balanced sample with Heckman sample selection correction**							
	0.046***	0.052***	0.072***	0.096***	0.100***	0.012	0.002
	(0.010)	(0.016)	(0.026)	(0.031)	(0.026)	(0.048)	(0.019)
No. of obs.	30867	9299	4301	5552	4014	1113	6588

Table 17. Associations between town-level production diversity and household dietary diversity (with different samples)

*** p<0.01, **p<0.05, * p<0.1. The p-values of coefficients are obtained from two-sided z-tests with standard errors clustered at the household level

	Correlated random effects models						
	All	Ethiopia	Malawi	Niger	Nigeria	Tanzania	Uganda
**(A) Farmer sample**							
	0.027***	0.058***	0.020	0.113***	0.056***	0.013	0.024
	(0.008)	(0.017)	(0.029)	(0.039)	(0.018)	(0.023)	(0.015)
No. of obs.	57175	9949	4125	5278	12681	10533	14609
**(B) Balanced sample**							
	0.034***	0.063***	0.038	0.079***	0.089***	0.021	0.034*
	(0.010)	(0.017)	(0.027)	(0.030)	(0.025)	(0.048)	(0.020)
No. of obs.	30867	9299	4301	5552	4014	1113	6588
**(C) Whole sample**							
	0.025***	0.054***	0.035	0.080***	0.067***	-0.000	0.015
	(0.007)	(0.016)	(0.026)	(0.030)	(0.015)	(0.021)	(0.014)
No. of obs.	65579	11410	4626	6536	15063	11830	16114
**(D) Farmer sample with Heckman sample selection correction**							
	0.027***	0.059***	0.016	0.114***	0.057***	0.011	0.024
	(0.008)	(0.017)	(0.029)	(0.039)	(0.018)	(0.023)	(0.015)
No. of obs.	57175	9949	4125	5278	12681	10533	14609
**(E) Balanced sample with Heckman sample selection correction**							
	0.031***	0.063***	0.039	0.073**	0.089***	0.003	0.011
	(0.010)	(0.017)	(0.028)	(0.030)	(0.025)	(0.047)	(0.020)
No. of obs.	30867	9299	4301	5552	4014	1113	6588

Table 18. Associations between district-level production diversity and household dietary diversity (with different samples)

*** p<0.01, **p<0.05, * p<0.1. The p-values of coefficients are obtained from two-sided z-tests with standard errors clustered at the household level

	Correlated random effects models						
	All	Ethiopia	Malawi	Niger	Nigeria	Tanzania	Uganda
**(A) Farmer sample**							
	0.028***	0.064***	-0.065	-0.025	0.100***	-0.018	0.019
	(0.010)	(0.018)	(0.044)	(0.050)	(0.023)	(0.028)	(0.018)
No. of obs.	57175	9949	4125	5278	12681	10533	14609
**(B) Balanced sample**							
	0.043***	0.070***	-0.042	0.028	0.165***	0.110**	0.052**
	(0.011)	(0.017)	(0.042)	(0.044)	(0.033)	(0.055)	(0.024)
No. of obs.	30867	9299	4301	5552	4014	1113	6588
**(C) Whole sample**							
	0.028***	0.058***	-0.043	0.028	0.111***	-0.035	0.015
	(0.009)	(0.016)	(0.042)	(0.044)	(0.020)	(0.026)	(0.017)
No. of obs.	65579	11410	4626	6536	15063	11830	16114
**(D) Farmer sample with Heckman sample selection correction**							
	0.028***	0.064***	-0.068	-0.023	0.100***	-0.018	0.022
	(0.010)	(0.018)	(0.044)	(0.050)	(0.023)	(0.028)	(0.018)
No. of obs.	57175	9949	4125	5278	12681	10533	14609
**(E) Balanced sample with Heckman sample selection correction**							
	0.044***	0.070***	-0.038	0.016	0.164***	0.116**	0.036
	(0.011)	(0.017)	(0.042)	(0.044)	(0.033)	(0.054)	(0.024)
No. of obs.	30867	9299	4301	5552	4014	1113	6588

Table 19. Definition and descriptive statistics of control variables

Name	Unit	Definition	Mean	SD
Household size	members	Number of household members	5.36	2.97
Share of dependent members	proportion	Share of dependent members per total household members	0.45	0.25
Head age	years	Age of household head	46.54	15.58
Head female	yes=1, no=0	Household head is female	0.25	0.43
Head literacy	yes=1, no=0	Household head can read and write	0.65	0.48
Motorbike	yes=1, no=0	Household has at least one motorbike	0.13	0.34
Phone	yes=1, no=0	Household has at least one phone	0.65	0.48
Electricity	yes=1, no=0	Household has electricity access	0.36	0.48
Wage employment	yes=1, no=0	Household has at least one member having wage employment	0.32	0.47
Nonfarm business	yes=1, no=0	Household has at least one member having nonfarm business	0.46	0.50
Weather shock	yes=1, no=0	Household suffered from at least one extreme weather event (e.g. drought, flood, hurricane) over the last 12 months	0.21	0.40
Farm land area	hectare	Farm-land area	1.40	5.43
Non-food cash crop	yes=1, no=0	Household has planted nonfood cash crop	0.19	0.39
Distance	km	Distance to the nearest urban center with more than 20,000 population	31.33	33.83

Table 20. Associations between farm-level production diversity (species count) and household dietary diversity (with different econometric models)

Robust standard errors in parentheses; *** p<0.01, **p<0.05, * p<0.1. For fixed-effects models, p-values are obtained from two-sided t-tests. For Poisson correlated random effects and correlated random effects models, p-values are obtained from two-sided z-tests

	All	Ethiopia	Malawi	Niger	Nigeria	Tanzania	Uganda
**Fixed-effects models**							
	0.048***	0.026***	0.084***	0.144***	0.045***	0.062***	0.047***
	(0.003)	(0.006)	(0.010)	(0.018)	(0.008)	(0.006)	(0.006)
No. of obs.	89742	13511	9163	7046	18592	21117	20313
**Poisson correlated random effects models**							
	0.046***	0.012**	0.094***	0.146***	0.050***	0.057***	0.050***
	(0.003)	(0.006)	(0.010)	(0.018)	(0.009)	(0.006)	(0.006)
No. of obs.	89742	13511	9163	7046	18592	21117	20313
**Correlated random effects models**							
	0.044***	0.017***	0.090***	0.140***	0.047***	0.058***	0.048***
	(0.003)	(0.006)	(0.010)	(0.018)	(0.008)	(0.006)	(0.005)
No. of obs.	89742	13511	9163	7046	18592	21117	20313

.

Table 21. Associations between farm-level production diversity (food groups) and household dietary diversity (with different econometric models)

Robust standard errors in parentheses; *** p<0.01, **p<0.05, * p<0.1. For fixed-effects models, p-values are obtained from two-sided t-tests. For Poisson correlated random effects and correlated random effects models, p-values are obtained from two-sided z-tests

	All	Ethiopia	Malawi	Niger	Nigeria	Tanzania	Uganda
**Fixed-effects models**							
	0.099***	0.058***	0.110***	0.252***	0.086***	0.147***	0.096***
	(0.005)	(0.011)	(0.015)	(0.035)	(0.014)	(0.011)	(0.010)
No. of obs.	89742	13511	9163	7046	18592	21117	20313
**Poisson correlated random effects models**							
	0.103***	0.048***	0.119***	0.250***	0.099***	0.145***	0.099***
	(0.005)	(0.011)	(0.014)	(0.035)	(0.015)	(0.011)	(0.010)
No. of obs.	89742	13511	9163	7046	18592	21117	20313
**Correlated random effects models**							
	0.100***	0.051***	0.116***	0.245***	0.095***	0.144***	0.097***
	(0.005)	(0.011)	(0.014)	(0.035)	(0.014)	(0.011)	(0.010)
No. of obs.	89742	13511	9163	7046	18592	21117	20313

Table 22. Associations between farm-level production diversity and household dietary diversity from own production (with different econometric models)

Robust standard errors in parentheses; *** p<0.01, **p<0.05, * p<0.1. For fixed-effects models, p-values are obtained from two-sided t-tests. For Poisson correlated random effects and correlated random effects models, p-values are obtained from two-sided z-tests

	All	Ethiopia	Malawi	Niger	Nigeria	Tanzania	Uganda
**Fixed-effects models**							
	0.232***	0.071***	0.314***	0.197***	0.233***	0.304***	0.227***
	(0.005)	(0.011)	(0.014)	(0.022)	(0.012)	(0.012)	(0.010)
No. of obs.	82550	10583	9155	7044	18429	17046	20293
**Poisson correlated random effects models**							
	0.202***	0.064***	0.291***	0.158***	0.220***	0.281***	0.229***
	(0.005)	(0.009)	(0.013)	(0.018)	(0.011)	(0.012)	(0.011)
No. of obs.	82550	10583	9155	7044	18429	17046	20293
**Correlated random effects models**							
	0.229***	0.073***	0.317***	0.197***	0.232***	0.295***	0.226***
	(0.005)	(0.011)	(0.013)	(0.022)	(0.012)	(0.012)	(0.010)
No. of obs.	82550	10583	9155	7044	18429	17046	20293

Table 23. Associations between farm-level production diversity and household dietary diversity from market purchases (with different econometric models)

Robust standard errors in parentheses; *** p<0.01, **p<0.05, * p<0.1. For fixed-effects models, p-values are obtained from two-sided t-tests. For Poisson correlated random effects and correlated random effects models, p-values are obtained from two-sided z-tests

	All	Ethiopia	Malawi	Niger	Nigeria	Tanzania	Uganda
**Fixed-effects models**							
	-0.002	0.040***	-0.016	0.236***	0.004	-0.007	-0.011
	(0.006)	(0.013)	(0.017)	(0.038)	(0.016)	(0.014)	(0.012)
No. of obs.	82550	10583	9155	7044	18429	17046	20293
**Poisson correlated random effects models**							
	0.013*	0.024	-0.010	0.236***	0.021	0.004	0.004
	(0.007)	(0.016)	(0.018)	(0.039)	(0.017)	(0.016)	(0.012)
No. of obs.	82550	10583	9155	7044	18429	17046	20293
**Correlated random effects models**							
	0.004	0.029**	-0.012	0.228***	0.015	-0.001	-0.005
	(0.006)	(0.013)	(0.017)	(0.038)	(0.016)	(0.014)	(0.012)
No. of obs.	82550	10583	9155	7044	18429	17046	20293

Table 24. Associations between farm-level production diversity and household dietary diversity (using the same sample for estimating village-, town-, and district-level production diversity) (with different econometric models)

Robust standard errors in parentheses; *** p<0.01, **p<0.05, * p<0.1. For fixed-effects models, p-values are obtained from two-sided t-tests. For Poisson correlated random effects and correlated random effects models, p-values are obtained from two-sided z-tests

	All	Ethiopia	Malawi	Niger	Nigeria	Tanzania	Uganda
**Fixed-effects models**							
	0.105***	0.061***	0.115***	0.248***	0.071***	0.193***	0.099***
	(0.006)	(0.011)	(0.020)	(0.036)	(0.016)	(0.014)	(0.011)
No. of obs.	65579	11410	4626	6536	15063	11830	16114
**Poisson correlated random effects models**							
	0.107***	0.051***	0.127***	0.244***	0.080***	0.192***	0.100***
	(0.006)	(0.011)	(0.019)	(0.036)	(0.016)	(0.014)	(0.011)
No. of obs.	65579	11410	4626	6536	15063	11830	16114
**Correlated random effects models**							
	0.105***	0.054***	0.124***	0.242***	0.080***	0.193***	0.099***
	(0.006)	(0.011)	(0.019)	(0.035)	(0.016)	(0.014)	(0.011)
No. of obs.	65579	11410	4626	6536	15063	11830	16114

Table 25. Associations between village-level production diversity and household dietary diversity (with different econometric models)

Robust standard errors in parentheses; *** p<0.01, **p<0.05, * p<0.1. For fixed-effects models, p-values are obtained from two-sided t-tests. For Poisson correlated random effects and correlated random effects models, p-values are obtained from two-sided z-tests

	All	Ethiopia	Malawi	Niger	Nigeria	Tanzania	Uganda
**Fixed-effects models**							
	0.028***	0.039**	0.058**	0.105***	0.053***	0.037*	0.008
	(0.007)	(0.015)	(0.026)	(0.031)	(0.016)	(0.019)	(0.014)
No. of obs.	65579	11410	4626	6536	15063	11830	16114
**Poisson correlated random effects models**							
	0.047***	0.052***	0.067***	0.114***	0.071***	0.057***	0.026*
	(0.007)	(0.015)	(0.026)	(0.031)	(0.016)	(0.019)	(0.013)
No. of obs.	65579	11410	4626	6536	15063	11830	16114
**Correlated random effects models**							
	0.044***	0.052***	0.066**	0.112***	0.067***	0.055***	0.020
	(0.007)	(0.015)	(0.026)	(0.031)	(0.016)	(0.019)	(0.013)
No. of obs.	65579	11410	4626	6536	15063	11830	16114

Table 26. Associations between town-level production diversity and household dietary diversity (with different econometric models)

Robust standard errors in parentheses; *** p<0.01, **p<0.05, * p<0.1. For fixed-effects models, p-values are obtained from two-sided t-tests. For Poisson correlated random effects and correlated random effects models, p-values are obtained from two-sided z-tests

	All	Ethiopia	Malawi	Niger	Nigeria	Tanzania	Uganda
**Fixed-effects models**							
	0.026***	0.059***	0.026	0.104***	0.062***	-0.009	0.006
	(0.007)	(0.016)	(0.027)	(0.031)	(0.016)	(0.021)	(0.014)
No. of obs.	65579	11410	4626	6536	15063	11830	16114
**Poisson correlated random effects models**							
	0.027***	0.051***	0.037	0.078***	0.069***	0.003	0.020
	(0.007)	(0.016)	(0.026)	(0.029)	(0.015)	(0.021)	(0.014)
No. of obs.	65579	11410	4626	6536	15063	11830	16114
**Correlated random effects models**							
	0.025***	0.054***	0.035	0.080***	0.067***	-0.000	0.015
	(0.007)	(0.016)	(0.026)	(0.030)	(0.015)	(0.021)	(0.014)
No. of obs.	65579	11410	4626	6536	15063	11830	16114

Table 27. Associations between district-level production diversity and household dietary diversity (with different econometric models)

Robust standard errors in parentheses; *** p<0.01, **p<0.05, * p<0.1. For fixed-effects models, p-values are obtained from two-sided t-tests. For Poisson correlated random effects and correlated random effects models, p-values are obtained from two-sided z-tests

	All	Ethiopia	Malawi	Niger	Nigeria	Tanzania	Uganda
**Fixed-effects models**							
	0.030***	0.062***	-0.046	0.059	0.129***	-0.019	0.003
	(0.009)	(0.016)	(0.042)	(0.045)	(0.021)	(0.026)	(0.018)
No. of obs.	65579	11410	4626	6536	15063	11830	16114
**Poisson correlated random effects models**							
	0.031***	0.055***	-0.040	0.031	0.107***	-0.038	0.020
	(0.009)	(0.017)	(0.043)	(0.046)	(0.020)	(0.026)	(0.018)
No. of obs.	65579	11410	4626	6536	15063	11830	16114
**Correlated random effects models**							
	0.028***	0.058***	-0.043	0.028	0.111***	-0.035	0.015
	(0.009)	(0.016)	(0.042)	(0.044)	(0.020)	(0.026)	(0.017)
No. of obs.	65579	11410	4626	6536	15063	11830	16114
