# Supplementary material for: Local and regional food production diversity are positively associated with household dietary diversity in rural Africa
Source: Nat Food. 2025 Jan 2;6(2):205–12. doi: 10.1038/s43016-024-01096-6 (PMC11850281; doi:10.1038/s43016-024-01096-6)
Supplement: Supplementary file 2 — Reporting Summary [file 43016_2024_1096_MOESM2_ESM.pdf]

nature portfolio

Corresponding author(s): Thanh-Tung Nguyen

Last updated by author(s): November 16, 2024

Reporting Summary

Nature Portfolio wishes to improve the reproducibility of the work that we publish. This form provides structure for consistency and transparency in reporting. For further information on Nature Portfolio policies, see our Editorial Policies and the Editorial Policy Checklist.

Statistics

For all statistical analyses, confirm that the following items are present in the figure legend, table legend, main text, or Methods section.

n/a	Confirmed	
☐	☒	The exact sample size (n) for each experimental group/condition, given as a discrete number and unit of measurement
☐	☒	A statement on whether measurements were taken from distinct samples or whether the same sample was measured repeatedly
☐	☒	The statistical test(s) used AND whether they are one- or two-sided<br>*Only common tests should be described solely by name; describe more complex techniques in the Methods section.*
☐	☒	A description of all covariates tested
☐	☒	A description of any assumptions or corrections, such as tests of normality and adjustment for multiple comparisons
☐	☒	A full description of the statistical parameters including central tendency (e.g. means) or other basic estimates (e.g. regression coefficient) AND variation (e.g. standard deviation) or associated estimates of uncertainty (e.g. confidence intervals)
☐	☒	For null hypothesis testing, the test statistic (e.g. F, t, r) with confidence intervals, effect sizes, degrees of freedom and P value noted<br>*Give P values as exact values whenever suitable.*
☒	☐	For Bayesian analysis, information on the choice of priors and Markov chain Monte Carlo settings
☒	☐	For hierarchical and complex designs, identification of the appropriate level for tests and full reporting of outcomes
☒	☐	Estimates of effect sizes (e.g. Cohen's d, Pearson's r), indicating how they were calculated

Our web collection on statistics for biologists contains articles on many of the points above.

Software and code

Policy information about availability of computer code

Data collection	We only use secondary data in this study. We did not collect data and did not use any software for data collection.
Data analysis	Data analysis is conducted by using the Stata software, version 16.0. https://doi.org/10.7910/DVN/NYPA0Z

For manuscripts utilizing custom algorithms or software that are central to the research but not yet described in published literature, software must be made available to editors and reviewers. We strongly encourage code deposition in a community repository (e.g. GitHub). See the Nature Portfolio guidelines for submitting code & software for further information.

Data

Policy information about availability of data

All manuscripts must include a data availability statement. This statement should provide the following information, where applicable:

- Accession codes, unique identifiers, or web links for publicly available datasets
- A description of any restrictions on data availability
- For clinical datasets or third party data, please ensure that the statement adheres to our policy

The data are publicly available for research purposes from https://www.worldbank.org/en/programs/lsms/initiatives/lsms-ISA

Research involving human participants, their data, or biological material

Policy information about studies with human participants or human data. See also policy information about sex, gender (identity/presentation), and sexual orientation and race, ethnicity and racism.

Reporting on sex and gender	N/A (we use secondary data)
Reporting on race, ethnicity, or other socially relevant groupings	N/A (we use secondary data)
Population characteristics	N/A (we use secondary data)
Recruitment	N/A (we use secondary data)
Ethics oversight	N/A (we use secondary data)

Note that full information on the approval of the study protocol must also be provided in the manuscript.

Field-specific reporting

Please select the one below that is the best fit for your research. If you are not sure, read the appropriate sections before making your selection.

☐ Life sciences ☒ Behavioural & social sciences ☐ Ecological, evolutionary & environmental sciences

For a reference copy of the document with all sections, see nature.com/documents/nr-reporting-summary-flat.pdf

Behavioural & social sciences study design

All studies must disclose on these points even when the disclosure is negative.

Study description	Study with observational, quantitative data and a longitudidal (panel) design. The data are evaluated with panel data models and correlated random effects (pseudo fixed effects) estimators.
Research sample	The data stem from the World Bank's Living Standards Measurement Study – Integrated Surveys on Agriculture (LSMS-ISA). The samples are nationally representative, including randomly selected households in six African countries. These countries were selected due to the availability of panel datasets and the widespread issues of undernutrition and low dietary quality.
Sampling strategy	We did not collect the data ourselves but use secondary LSMS-ISA data. The data were collected by the official statistical offices in the study countries with support from the World Bank as part ofthe larger LSMS-ISA initiative. Households were sampled using the stratified random sampling procedure. First, primary sampling units (PSU) within major zone strata selected based on the national population frame, followed by the second stage that households are randomly selected. Detailed information on the sampling procedure in each country is provided in https://www.worldbank.org/en/programs/lsms/initiatives/lsms-ISA
Data collection	We did not collect the data ourselves but use secondary LSMS-ISA data. In all countries, the LSMS-ISA data were collected through face-to-face interviews using structured questionnaires.
Timing	The data in the six study countries were collected in multiple survey waves. In Ethiopia, 3 waves (09/11-03/12, 09/13-04/14, 09/15-04/16), in Malawi, 4 waves (03/10-03/11, 04/13-11/13, 04/16-04/17, 04/19-03/20), in Niger, 2 waves (07/11-01/12, 09/14-03/15), in Nigeria, 4 waves (08/10-04/11; 09/12-04/13, 08/15-04/16; 07/18-02/2019), in Tanzania, 5 waves (10/08-09/09, 10/10-11/11, 10/12-11/13, 10/14-10/15, 01/19-01/22), and in Uganda, 7 waves (09/09-08/10, 11/10-10/11, 11/11-12/12, 09/13-08/14, 03/15-03/16, 03/18-02/19, 03/19-02/20) are available.
Data exclusions	We use the data as publicly provided on the LSMS-ISA website. All survey waves from the six countries combined include around 90,000 observations. We excluded observations with missing values or outliers for food consumption and other key variables. Our final sample includes 89,742 observations
Non-participation	Only households that agreed to be surveyed were included (https://www.worldbank.org/en/programs/lsms/inititiatives/lsms-ISA)
Randomization	Data were collected through stratified random sampling. The data are observational, no interventions were conducted.

Reporting for specific materials, systems and methods

We require information from authors about some types of materials, experimental systems and methods used in many studies. Here, indicate whether each material, system or method listed is relevant to your study. If you are not sure if a list item applies to your research, read the appropriate section before selecting a response.

Materials & experimental systems

n/a	Involved in the study
☒	☐ Antibodies
☒	☐ Eukaryotic cell lines
☒	☐ Palaeontology and archaeology
☒	☐ Animals and other organisms
☒	☐ Clinical data
☒	☐ Dual use research of concern
☒	☐ Plants

Methods

n/a	Involved in the study
☒	☐ ChIP-seq
☒	☐ Flow cytometry
☒	☐ MRI-based neuroimaging

Plants

Seed stocks	N/A
Novel plant genotypes	N/A
Authentication	N/A
